# Apolipoprotein E promotes primary resistance to AR-targeted therapy via inducing TRIM25-mediated AR ubiquitination and sensitizes immunotherapy in prostate cancer

**DOI:** 10.7150/thno.109994

**Published:** 2025-04-21

**Authors:** Chaofan Liu, Xi Wang, Qinyu Li, Xintao Gao, Kai Zeng, Beining Li, Jianping Miao, Bolong Zheng, Jihong Liu, Zhihua Wang, Xianglin Yuan, Bo Liu

**Affiliations:** 1 Department of Oncology, Tongji Hospital, Tongji Medical College, Huazhong University of Science and Technology, Wuhan 430030, Hubei, China.; 2 Department of Urology, Sir RunRun Shaw Hospital, College of Medicine, Zhejiang University, Hangzhou, China.; 3 Department of Urology, Tongji Hospital, Tongji Medical College, Huazhong University of Science and Technology, Wuhan 430030, Hubei, China.; 4 Department of Geriatrics, Tongji Hospital, Tongji Medical College, Huazhong University of Science and Technology, Wuhan, Hubei, China.; 5 School of Computer Science and Technology, Huazhong University of Science and Technology, China.

**Keywords:** Apolipoprotein E, AR-targeted therapy, immunotherapy, precision therapy, prostate cancer

## Abstract

**Rationale:** Prostate cancer (PCa) growth is facilitated by the androgen receptor (AR) and its downstream signaling pathways, making AR-targeted therapy crucial for treating advanced stages. Despite this, the response to AR-targeted therapies is inconsistent, with a significant proportion of patients even exhibiting unresponsiveness to therapy from the outset, known as primary resistance. Therefore, a refined categorization framework is imperative for the timely detection of resistant phenotypes and the exploration of novel therapeutic avenues.

**Methods:** Tissue microarrays and clinical cohorts were employed to delineate the impact of APOE on the prognostic outcomes and therapeutic resistance in PCa patients. Employing flow cytometry, immunoprecipitation, and mass spectrometry, we dissected the molecular underpinnings of APOE's role in conferring resistance to AR-targeted interventions. Single-cell RNA sequencing elucidated the intricate transcriptomic profiles of PCa with elevated APOE expression. Additionally, the therapeutic potential of anti-PD-L1 agents in treating PCa with APOE induction was rigorously assessed.

**Results:** In this study, we elucidated the pivotal role of APOE in mediating primary resistance to AR-targeted therapy in PCa through the suppression of AR signaling pathways. Mechanistically, APOE was found to enhance the ubiquitination and subsequent degradation of AR by mediating the interaction between the E3-ligase TRIM25 and AR, concurrently dampening the transcriptional activity of AR. Additionally, elevated APOE expression was correlated with an augmented response to anti-PD-L1 treatment, hinting at the therapeutic advantage of immunotherapy in APOE-high PCa contexts.

**Conclusions:** APOE expression could serve as a prognostic biomarker, pivotal for forecasting responses to both AR-targeted therapy and immunotherapy, thereby offering an innovative strategy for the personalized selection of treatment modalities in PCa.

## Introduction

Prostate cancer (PCa), the most frequently diagnosed malignancy in men, is largely regulated by androgen sensitivity 1. Endocrine therapies exploit this dependency by either diminishing the endogenous androgen synthesis or by targeting the androgen receptor (AR) directly 2-4. Despite significant advancements in therapeutic approaches, the cornerstone of advanced PCa treatment remains AR-targeted therapy, encompassing androgen deprivation therapy (ADT) and anti-androgenic therapy 5,6. Nevertheless, the duration to biochemical recurrence and the efficacy of anti-androgenic drugs exhibits notable heterogeneity among patients. A considerable subset of individuals may even demonstrate primary resistance to the treatment 7. Hence, it is of utmost significance to establish validated and clinically approved predictive biomarkers that can effectively discern patients who will benefit from AR-targeted therapy.

Apolipoprotein E (APOE), a polymorphic protein, plays a multifaceted role in various biological processes 8. It is primarily involved in the transportation of lipids, metabolism of lipoproteins, and modulation of a spectrum of cellular physiological and pathological activities, including the regulation of inflammatory responses and immune functions 9-11. Recently, APOE has been linked to various malignancies, establishing a complex relationship with tumorigenesis 12-15. APOE is implicated not only in tumor metabolism and immune modulation but also in tumor invasion and metastasis 8. Bancaro et al. demonstrated the upregulation of APOE and TREM2 expression in PCa, which exhibits a significant association with unfavorable prognostic outcomes 16. However, further research is necessary to elucidate the potential role of APOE in therapeutic strategies for PCa. In this study, we have elucidated that APOE induced primary resistance to AR-targeted therapies in PCa by promoting ubiquitination and degradation of AR. Furthermore, our research revealed that elevated levels of APOE expression were associated with a significant decrease in the prevalence of myeloid-derived suppressor cells (MDSCs) and an enhancement in the infiltration of CD8+ cytotoxic T cells. This alteration ultimately contributed to an enhanced response to immunotherapy for APOE^high^ PCa.

## Results

### APOE was upregulated in PCa and predicted poor prognosis

**Figure 1:**
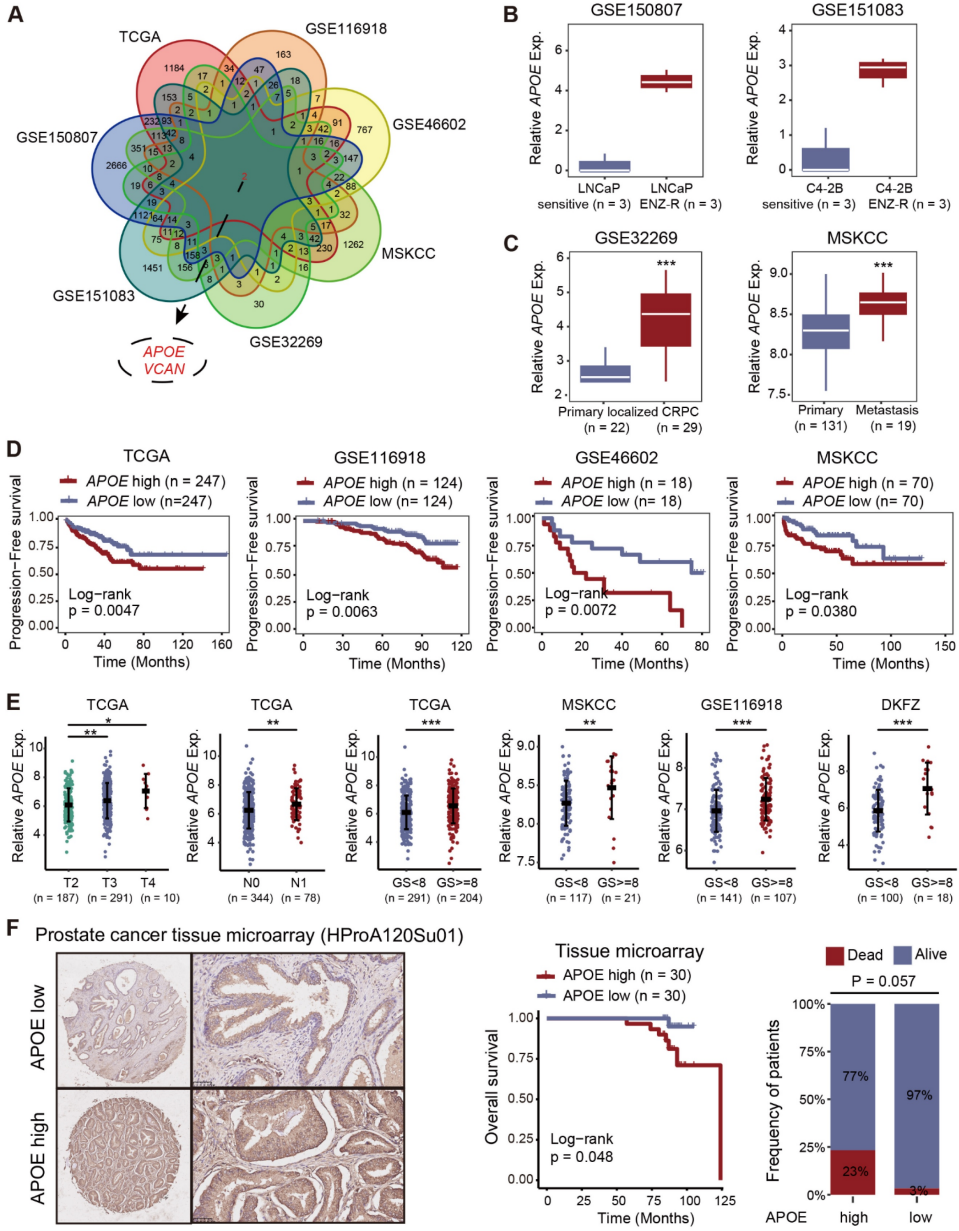
**Elevated APOE levels indicate adverse prognosis in PCa. (A)** Two pivotal genes, APOE and VCAN, were identified through rigorous screening of substantial genes instrumental in the tumorigenesis and progression of PCa, across seven separate PCa datasets. **(B)** Comparative analysis of APOE expression in LNCaP sensitive versus LNCaP ENZ-R cells (GSE150807); and C4-2B sensitive versus C4-2B ENZ-R cells (GSE151083). (C) Comparative expression levels of APOE in primary PCa and CRPC samples (GSE32269); and primary and metastatic PCa (MSKCC). **(D)** Utilizing Kaplan-Meier methods, the correlation between APOE gene expression and patient PFS was examined across TCGA, GSE116918, GSE46602, and MSKCC databases. **(E)** Increased APOE expression was closely associated with higher pathological T stage, N stage, and Gleason score. **(F)** Evaluation of APOE protein expression in PCa tissue microarrays (HProA120Su01) showed that patients with higher APOE protein levels had poorer OS than those in the low APOE group. *, p < 0.05; **, p < 0.01; ***, p < 0.001.

To screen significant genes with crucial roles in tumorigenesis and AR-targeted therapy resistance in PCa, we conducted a comprehensive analysis of transcriptome data from seven datasets (TCGA, GSE116918, GSE46602, MSKCC, GSE32269, GSE151083, and GSE150807). We focused on genes closely associated with prognosis (TCGA, GSE116918, GSE46602, and MSKCC) and differentially expressed genes between primary localized PCa and castration-resistant prostate cancer (CRPC) (GSE32269), as well as between enzalutamide-sensitive and -resistant PCa cell lines (GSE151083 and GSE150807), to refine the list of candidate genes (**Figure 1A and Table S1-7**). Ultimately, we identified two genes of interest, APOE and VCAN. Our analysis revealed that although VCAN was downregulated in tumor tissues, it paradoxically emerged as a prognostic risk factor for PCa. This inconsistency led us to deprioritize further investigation (**Figure S1A-B**). In contrast, APOE exhibited significant upregulation in both PCa tissues (**Figure S1C**) and enzalutamide-resistant PCa cell lines (**Figure 1B**). Furthermore, APOE expression levels in metastatic PCa and CRPC were consistently higher compared to those in primary localized PCa (**Figure 1C**). The Kaplan-Meier survival analysis demonstrated that elevated APOE expression was associated with poorer progression-free survival (PFS) in multiple datasets (**Figure 1D and Figure S1D**). In subsequent analysis, we investigated the correlation between APOE expression levels and various clinicopathological parameters in PCa. High APOE expression was found to be positively associated with advanced tumor stage and higher Gleason scores across multiple cohorts (**Figure 1E and Figure S1E**). To substantiate the clinical relevance of APOE, immunohistochemistry (IHC) was employed to assess APOE protein levels in a PCa tissue microarray (HProA120Su01). The findings indicated that patients with elevated APOE protein levels experienced significantly reduced overall survival (**Figure 1F**). These findings implied that APOE was implicated in the progression of PCa and may serve as an indicator of a less favorable prognosis for PCa patients.

### APOE mediated AR-targeted therapy resistance

**Figure 2:**
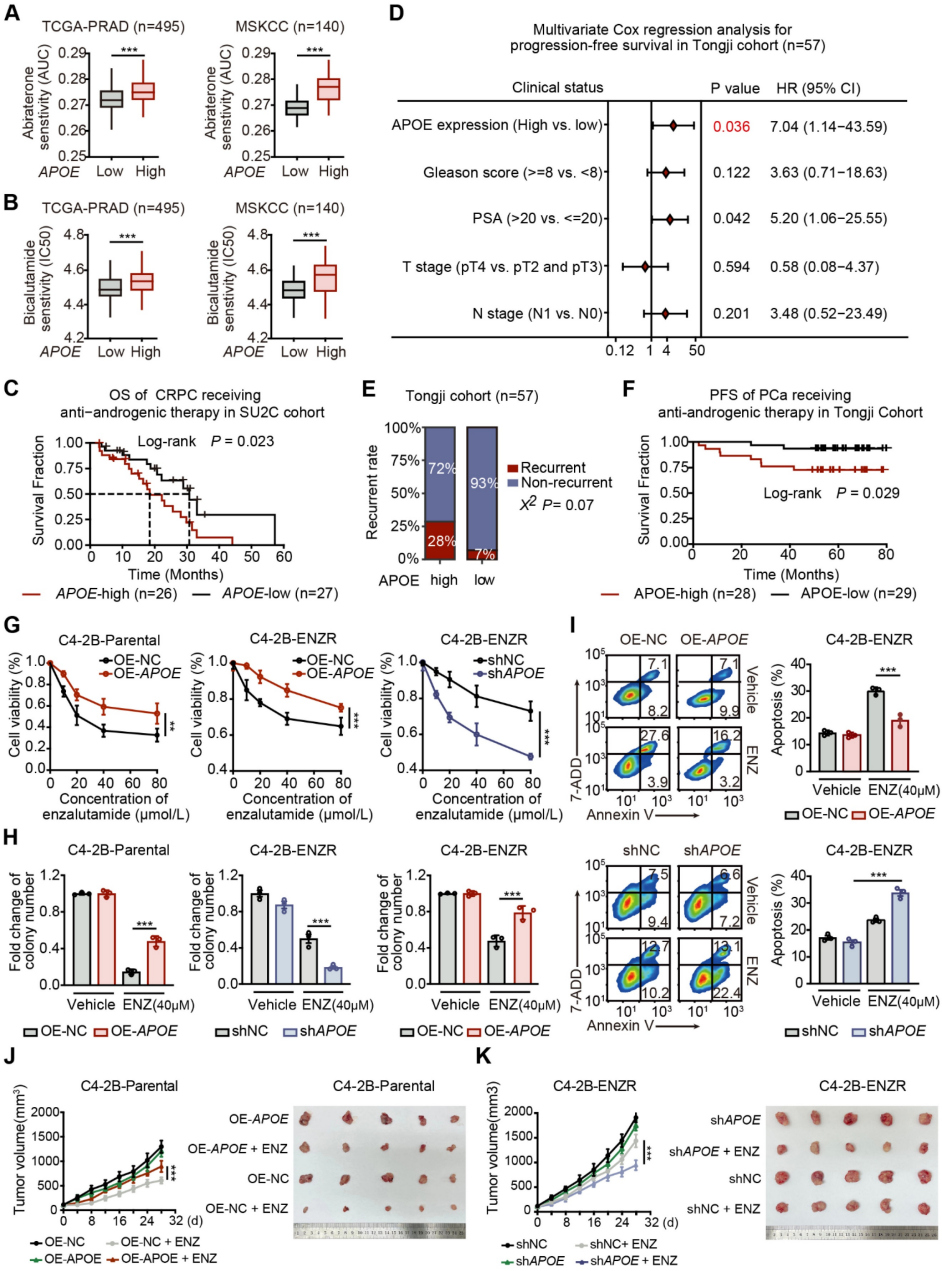
**APOE promotes resistance to AR-targeted treatment. (A)** Differential Abiraterone sensitivity (AUC) between high and low APOE expression groups was evaluated using TCGA-PRAD and MSKCC cohorts. **(B)** Bicalutamide sensitivity (IC50) was compared between the high and low APOE expression groups using the TCGA-PRAD and MSKCC cohorts. **(C)** Kaplan-Meier survival curve demonstrated a poorer prognosis for APOE-high patients post Anti-androgenic therapy, compared with APOE-low patients among the 53 CRPC patients in the SU2C cohort. **(D)** Multivariate Cox regression analyses attest to APOE expression as an independent prognostic variable for PCa in the Tongji PCa cohort. **(E)** The APOE-high group displayed a significantly higher recurrence rate compared to the APOE-low group. **(F)** In the Tongji PCa cohort, the log-rank test indicated that patients with high APOE expression exhibited poorer prognosis than those with low APOE expression. **(G)** Cell viability in the indicated cell lines under ENZ treatment was assayed using CCK8. **(H)** ENZ-treated C4-2B-ENZR cell proliferation was quantified using colony formation assays. **(I)** After transfection, the apoptosis rate in ENZ-treated C4-2B-ENZR cells was assessed through flow cytometry analysis. **(J-K)** APOE expression influenced xenograft PCa tumor growth. C4-2B-Parental and C4-2B-ENZR cells, post-transfection, were subcutaneously implanted into nude mice; resultant tumors from euthanized mice were then compared between groups. *, p < 0.05; **, p < 0.01; ***, p < 0.001.

Given the considerable upregulation of APOE in CRPC and enzalutamide-resistant PCa cell lines, we therefore explored the potential association between APOE expression and resistance to AR-targeted therapy in PCa. We leveraged the CPG and PRISM databases to assess the sensitivity of bicalutamide and abiraterone to varying levels of APOE expression. The findings revealed a substantial decrease in the responsiveness to AR-targeted therapy in patients exhibiting elevated APOE levels (**Figure 2A-B**). Consistent with these results, survival data from the Su2c cohort of CRPC patients undergoing anti-androgenic therapy showed that the group with high APOE expression had a significantly shorter overall survival span (**Figure 2C**).

To further validate our findings, we conducted an assessment within our Tongji PCa cohort to determine the relationship between survival outcomes and APOE expression levels. A multivariate Cox regression analysis was employed to ascertain the prognostic significance of APOE expression alongside other risk factors such as Gleason score, PSA levels, and pathological T and N stages. The analysis confirmed that elevated APOE protein levels were an independent predictor of poor prognosis in PCa patients (**Figure 2D**). Additionally, a higher recurrence rate was noted in the group with high APOE expression (**Figure 2E**), and survival analysis indicated that increased APOE protein expression was associated with inferior PFS in patients who received anti-androgenic therapy post-standard surgery (**Figure 2F**).

To corroborate our findings, we constructed the enzalutamide resistant C4-2B (C4-2B-ENZR) cells by subjecting C4-2B cells to escalating doses of enzalutamide, starting at 5 µM and incrementally increasing to 10 µM over a 16-week period (**Figure S2A**). Subsequently, we overexpressed APOE in C4-2B-Parental and C4-2B-ENZR cells, while shRNA was utilized to achieve stable depletion of APOE in C4-2B-ENZR cells. Cell viability and colony formation assays revealed that increased APOE expression mitigated the suppressive effects of enzalutamide on both C4-2B-Parental and C4-2B-ENZR cells. Conversely, APOE depletion exacerbated the inhibitory effects of enzalutamide on C4-2B-ENZR cells (**Figure 2G-H**). Consistently, overexpression of APOE reduced the apoptosis rate of C4-2B-ENZR cells treated with enzalutamide, whereas APOE silencing produced the opposite effect** (Figure 2I)**. Moreover, *in vivo* studies revealed that APOE overexpression significantly conferred resistance to enzalutamide (**Figure 2J**), whereas APOE depletion sensitized C4-2B-ENZR tumors to enzalutamide therapy (**Figure 2K**). Collectively, these findings suggest that APOE overexpression mediates primary resistance to AR-targeted therapy, and that downregulation of APOE may enhance sensitivity to AR-targeted therapy in PCa.

### APOE binds to AR and promotes its ubiquitination-mediated degradation

**Figure 3:**
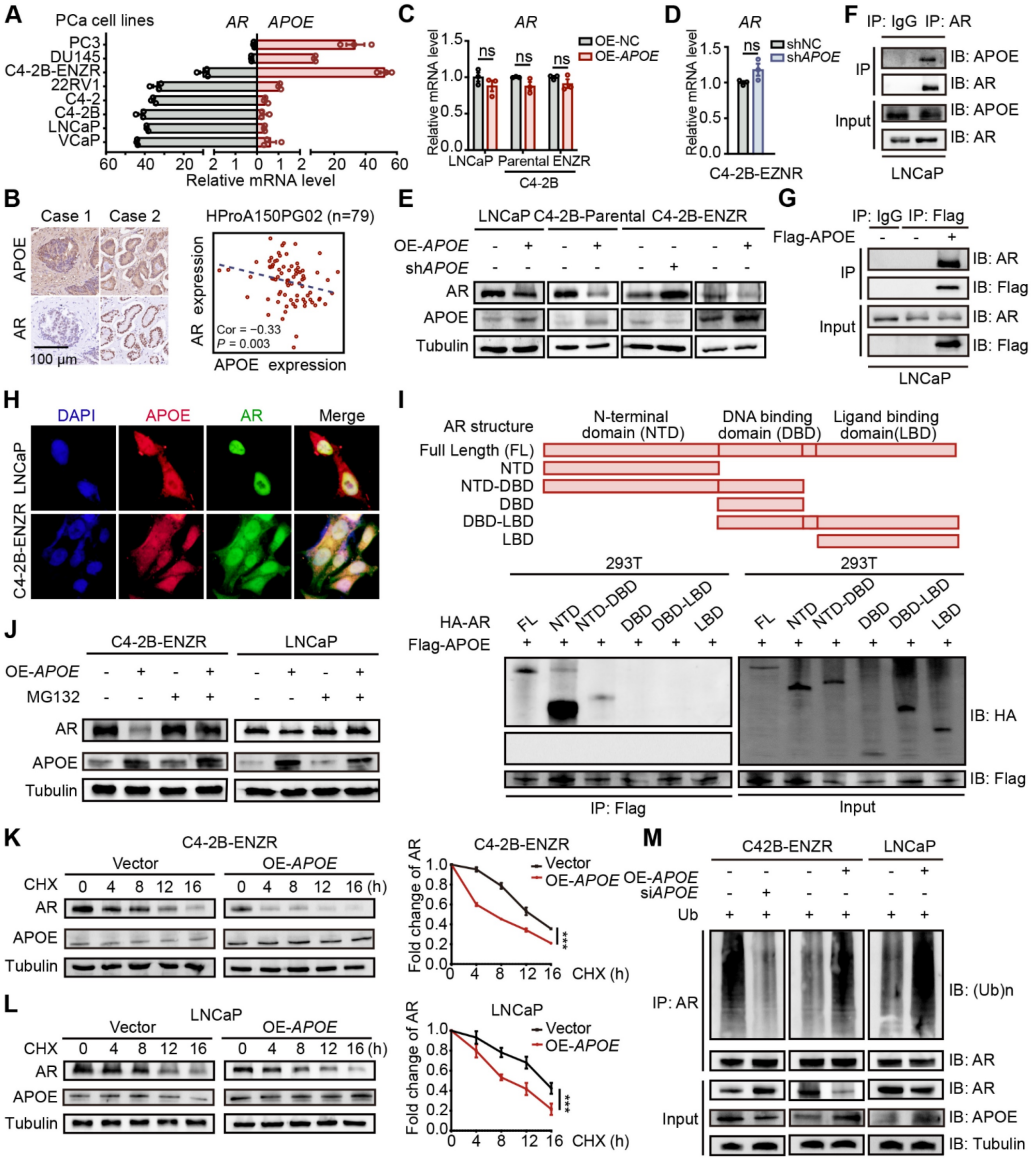
** APOE binds to AR and promotes its ubiquitination-mediated degradation. (A)** Analysis of relative mRNA levels of APOE and AR in various PCa cell lines. APOE mRNA expression was significantly upregulated in androgen-independent PCa cell lines. **(B)** An inverse relationship was found between APOE and AR protein expression within the PCa tissue array (HProA150PG02). **(C)** The mRNA levels of AR were not significantly affected by the overexpression of APOE in LNCaP, C4-2B-Parental, and C4-2B-ENZR cell lines. **(D)** mRNA levels of AR in C4-2B-ENZR cells remained largely unchanged after APOE depletion. **(E)** Western blot analysis indicates AR protein expression in PCa cell lines following overexpression or depletion of APOE. **(F-G)** Co-IP assays were carried out to demonstrate the interaction between APOE and AR in LNCaP cells. **(H)** Colocalization of APOE and AR was demonstrated via immunofluorescence in LNCaP and C4-2B-ENZR cells. **(I)** Co-IP assays were executed on lysed HEK293T cells previously transfected with varying HA-labeled AR plasmids (HA-AR, HA-AR-NTD, HA-AR-DBD, HA-AR-LBD, HA-AR-NTD-DBD, and HA-AR-DBD-LBD). **(J)** LNCaP and C4-2B-ENZR cells overexpressing APOE or the control were subjected to MG132 treatment followed by immunoblotting for AR detection. **(K-L)** After CHX treatment, APOE-overexpressing or control LNCaP (L) and C4-2B-ENZR (K) cells were collected at time intervals of 0, 4, 8, 12, and 16 h for evaluation of AR protein levels by immunoblotting. **(M)** LNCaP and C4-2B-ENZR cells with APOE overexpression or depletion were treated with MG132 and subjected to AR antibody immunoprecipitation, followed by immunoblotting. ***, p < 0.001.

Anti-androgenic therapies aim to inhibit PCa cell growth and survival by targeting AR signaling, thereby positioning AR expression as a critical factor influencing resistance to AR-targeted therapies. In light of this, we examined the relationship between AR and APOE expression levels. Interestingly, the C4-2B-ENZR cell line, along with two well-characterized enzalutamide-resistant PCa cell lines, DU145 and PC3, displayed increased APOE expression concomitant with diminished AR expression relative to sensitive cell lines (**Figure 3A**). Additionally, an analysis of tumor tissue from a PCa tissue array disclosed an inverse correlation between APOE and AR protein expression (**Figure 3B**). These findings suggested a possible regulatory role of APOE in modulating AR expression. However, despite alterations in APOE levels across different PCa cell lines, no substantial variations were noted in AR mRNA levels (**Figure 3C-D**). Conversely, an immunoblotting analysis revealed a diminished AR protein quantity in cells with APOE overexpression and a converse effect in cells with APOE suppression (**Figure 3E**), indicating that APOE regulated AR expression at the post-translational level. Subsequently, co-immunoprecipitation (Co-IP) experiments were further executed to investigate the interaction between APOE and AR (**Figure 3F-G**), and the immunofluorescence co-localization assay also verified this interplay (**Figure 3H**).

Given the structural composition of the AR, which encompasses three functional domains (the N-terminal domain (NTD), DNA binding domain (DBD), ligand binding domain (LBD) and a hinge link DBD and LBD), we generated truncated AR mutants corresponding to these domains. Utilizing a Co-IP assay in HEK293T cells, we aimed to pinpoint the domain responsible for the interaction with APOE. The findings indicated that the NTD and the fusion of NTD and DBD, rather than the DBD or LBD alone, were capable of binding to Flag-APOE, thereby identifying the NTD as the critical region mediating the APOE-AR interaction (**Figure 3I**). Given that APOE has been shown to influence the amount of AR protein, we hypothesized that APOE may participate in the regulation of AR stability through the ubiquitin-proteasome system, which has been extensively documented to be involved in AR modification. Immunoblotting analysis revealed that the proteasome inhibitor MG132 significantly counteracted the AR protein reduction caused by APOE overexpression (**Figure 3J**). Furthermore, the cycloheximide (CHX) chase assay indicated that APOE overexpression substantially decreased the half-life of the AR protein in both LNCaP and C4-2B-ENZR cells (**Figure 3K-L**). Additionally, APOE overexpression led to increased AR ubiquitination in C4-2B-ENZR and LNCaP cells, while APOE knockdown had the opposite effect (**Figure 3M**). Taken together, these findings suggest that APOE interacts with AR, promoting its ubiquitination and subsequent proteasomal degradation.

### APOE promotes TRIM25-mediated AR ubiquitination at K311

**Figure 4:**
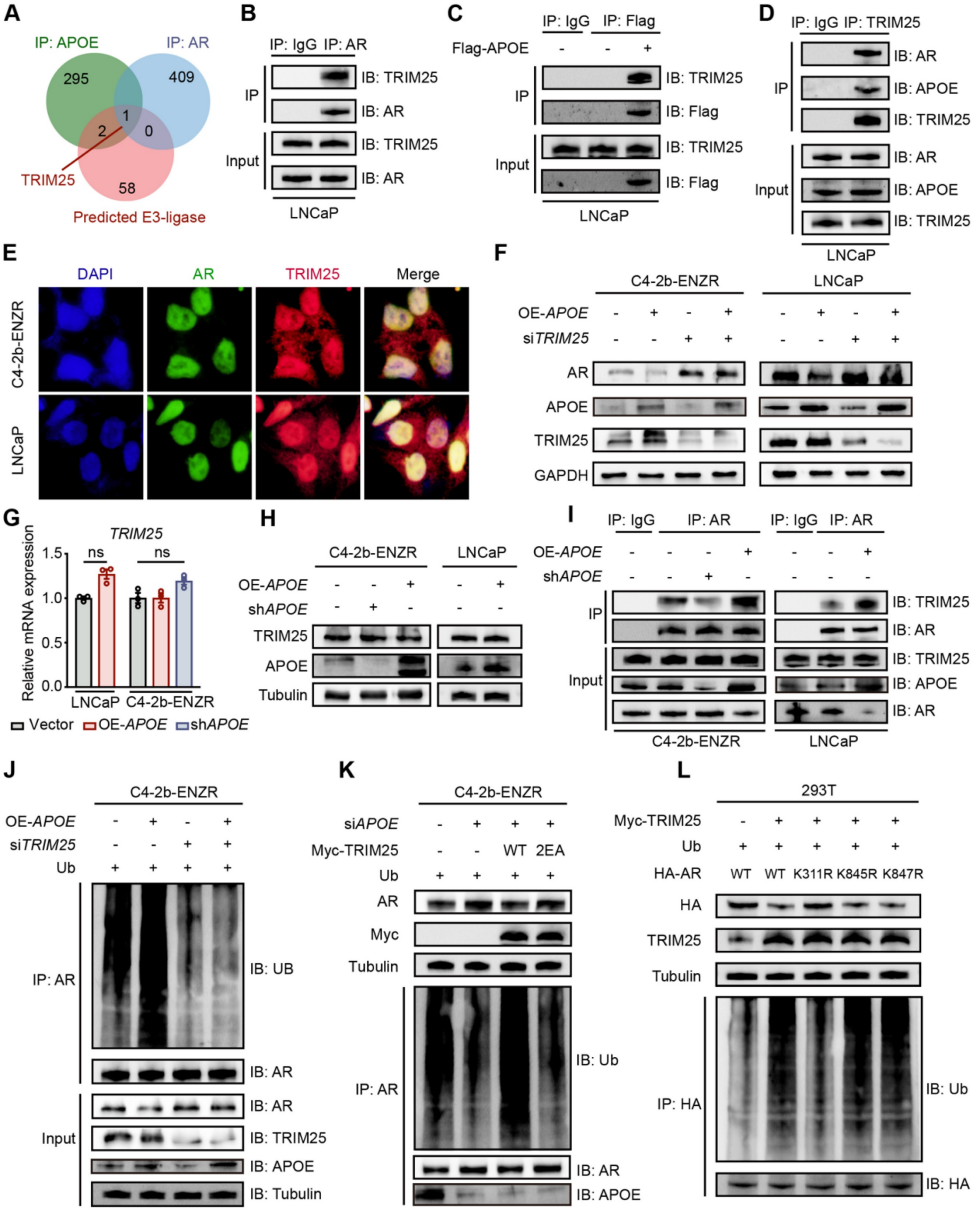
** APOE facilitates TRIM25-mediated AR ubiquitination at K311. (A)** Venn diagram highlights common binding proteins of APOE and AR. These proteins were concurrently identified as E3 ligases targeting AR poly-ubiquitination by Ubibrowser v2. **(B-D)** Co-IP assays were conducted in LNCaP cells to examine the interaction between TRIM25 and APOE/AR. **(E)** Immunofluorescence reveals colocalization of TRIM25 and AR in LNCaP and C4-2B-ENZR cells. **(F)** After LNCaP and C4-2B-ENZR cells overexpressing APOE were treated with siTRIM25 or a control, Western blotting was performed to determine AR and TRIM25 protein levels. **(G-H)** mRNA (G) and protein (H) levels of TRIM25 were assessed in LNCaP and C4-2B-ENZR cells treated with either siAPOE or an APOE expression vector. **(I)** Following transfection with siRNA against APOE or APOE expression vector in LNCaP and C4-2B-ENZR cells, Co-IP with AR antibody was conducted, followed by Western blotting using TRIM25 and AR antibodies. **(J)** C4-2B-ENZR cells were transfected with the indicated siRNA and expression vectors along with a ubiquitin plasmid. Protein lysates from these cells underwent Co-IP with AR antibody, followed by Western blotting using the indicated antibodies. **(K)** After transfecting C4-2B-ENZR cells with APOE siRNA alone or in combination with TRIM25 wild-type or mutant plasmid, Western blotting was performed to measure AR and TRIM25 protein levels. Protein lysates also underwent Co-IP with an AR antibody, followed by Western blotting with specified antibodies. **(L)** In HEK293T cells transfected with a TRIM25 expression vector in combination with an AR wild-type or mutant plasmid (K311R, K845R, and K847R), Western blotting was conducted to determine AR and TRIM25 protein levels. Protein lysates also underwent Co-IP with an HA antibody, followed by Western blotting with the indicated antibodies.

To elucidate the mechanism by which APOE regulates AR protein ubiquitination, we conducted mass spectrometry analysis to identify the specific E3 ligase responsible for poly-ubiquitination of AR in the context of APOE-induced AR destabilization. By combining the mass spectrometry data with the predictions from the Ubibrowser v2 online tool, we discovered that TRIM25 was a candidate protein that interacted with both APOE and AR and may play a role in AR ubiquitination (**Figure 4A**). TRIM25, a member of the tripartite motif (TRIM) family known for its E3 ubiquitin ligase activity, was found to be associated with poor prognosis in PCa patients with high mRNA expression levels, as indicated by Kaplan-Meier survival curves across various cohorts (**Figure S3**). Co-IP experiments confirmed the interaction among AR, APOE, and TRIM25 (**Figure 4B-D**). Additionally, colocalization of TRIM25 with AR was observed in both LNCaP and C4-2B-ENZR cells (**Figure 4E**). According to the results of rescue experiments, OE-APOE-induced AR degradation was abrogated by using siTRIM25 in LNCaP and C4-2B-ENZR cells, indicating that TRIM25 played pivotal roles in APOE-mediated AR destabilization (**Figure 4F**). Surprisingly, APOE interference or overexpression did not change the mRNA expression or protein abundance of TRIM25 (**Figure 4G-H**). However, co-IP assays revealed that the interaction between TRIM25 and AR was enhanced in cells with APOE overexpression and diminished in cells with APOE knockdown (**Figure 4I**), indicating that APOE mainly regulated the recruitment of TRIM25 to AR. Moreover, the increase in AR ubiquitination levels mediated by APOE overexpression was completely negated by TRIM25 knockdown (**Figure 4J**). Similarly, the suppression of AR ubiquitination levels by APOE knockdown was rescued by the overexpression of wild-type TRIM25 but not by a loss-of-function mutant (Glu9 and Glu10 mutated to Ala, termed TRIM25-2EA) (**Figure 4K**). This finding underscored the indispensable role of TRIM25 in mediating the regulatory effects of APOE on AR.

AR possesses three lysine residues, K311, K845, and K847, which are susceptible to poly-ubiquitination, leading to its degradation. We constructed three AR mutants with lysine 311, 845 and 847 mutated to arginine (termed K311R, K845R and K847R). To pinpoint the ubiquitination sites, we conducted Co-IP assays, which indicated that the K311R mutation was the only one that disrupted TRIM25-mediated AR ubiquitination (**Figure 4L**). Altogether, these findings illustrated that APOE facilitated the poly-ubiquitination of AR at lysine 311 through enhancing the interaction between TRIM25 and AR.

### APOE suppresses AR transcription activity

**Figure 5:**
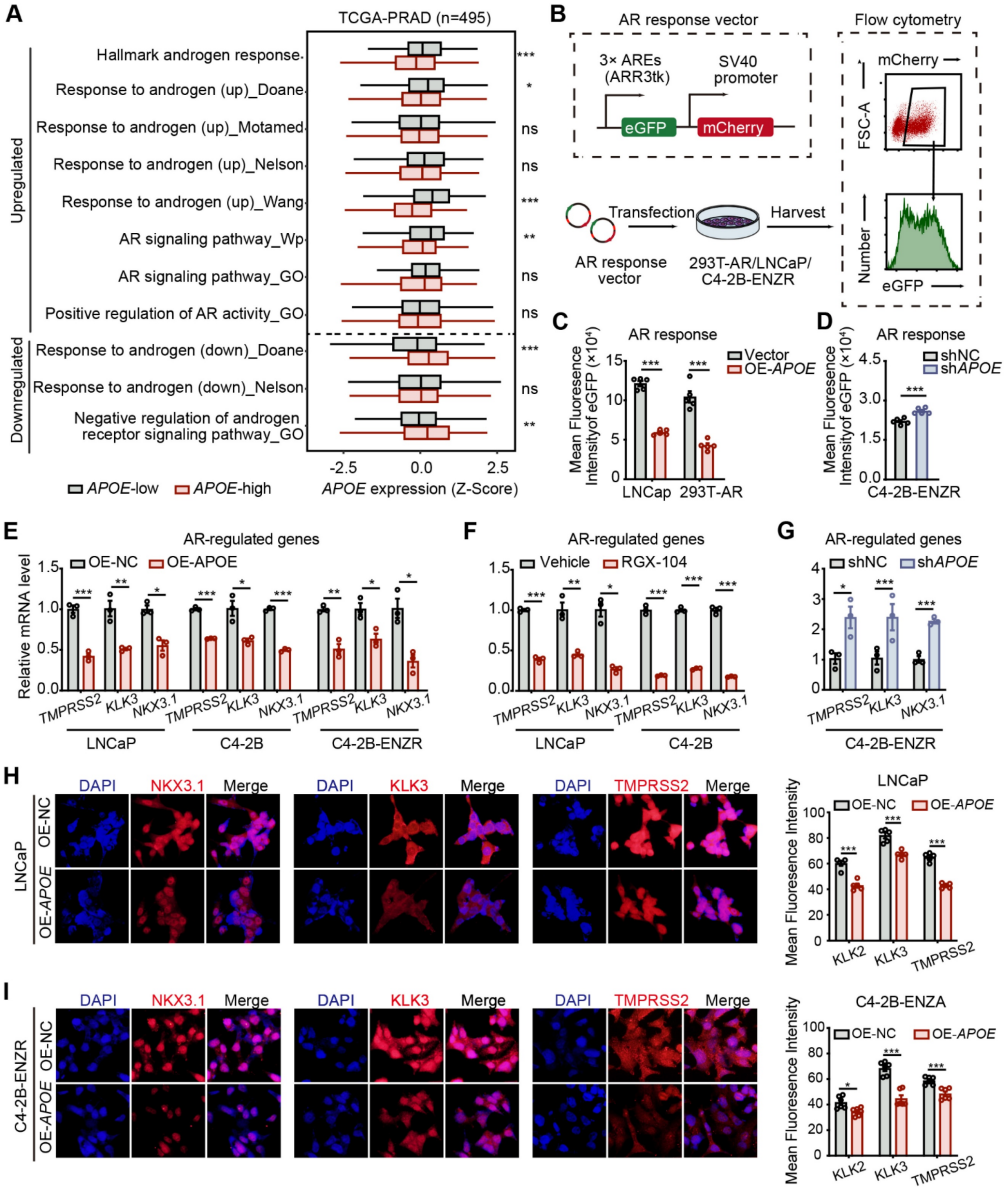
** APOE suppresses AR transcription activity. (A)** GSVA scores comparing the androgen response gene signatures and AR signaling pathway between the APOE-high and APOE-low groups. **(B)** The 293T (transfected with AR), LNCaP, and C4-2B-ENZR reporter cell lines were generated by transfection with the eGFP AR reporter vector. Cells successfully transfected were positively sorted based on mCherry expression via flow cytometry. Subsequently, their AR activities were sorted depending on eGFP AR-reporter expression. **(C-D)** Mean fluorescence intensity (MFI) of eGFP in reporter cell lines where APOE was overexpressed (B) and reduced (C). **(E-G)** qRT-PCR analysis illustrating the expression levels of AR-targeted genes (TMPRSS2, KLK3, NKX3.1) in specified PCa cell lines overexpressing APOE (H), treated with the APOE agonist RGX-104 (I), or with reduced APOE levels (J). **(H-I)** Representative images of immunofluorescence staining of AR-targeted genes (TMPRSS2, KLK3, NKX3.1) in LNCaP (K) and C4-2B-ENZR (L) cells overexpressing APOE. *, p < 0.05; **, p < 0.01; ***, p < 0.001.

The preceding findings indicated that APOE has an affinity for the NTD of AR, which is instrumental in AR's transcriptional capabilities 17. It is worth noting that prior research has demonstrated a strong association between AR activity and the responsiveness of PCa to AR-targeted therapy. Specifically, it has been observed that tumors with diminished AR activity exhibit a primary resistance to androgen deprivation therapy 7,18. Consequently, we utilized the "ssGSEA" algorithm to assess the enrichment scores of gene sets associated with androgen responsiveness and AR activity. Our results revealed a significant link between elevated APOE levels and attenuated androgen response as well as AR activity (**Figure 5A**). To directly assess the influence of APOE on AR functionality, we utilized an AR response reporter to gauge AR transcriptional activity. This reporter, containing three AR binding motifs that drive the expression of eGFP, was transiently introduced into HEK293T cells, co-transfected with an AR expression vector, as well as into LNCaP and C4-2B-ENZR cells. The transcriptional activity of AR, reflected by eGFP fluorescence intensity, was measured using flow cytometry (**Figure 5B**). The mean fluorescence intensity (MFI) of eGFP in HEK293T and LNCaP cells was found to diminish with APOE overexpression (**Figure 5C**). In contrast, a noticeable increase in MFI was observed in C4-2B-ENZR cells following the introduction of siAPOE (**Figure 5D**), indicating a regulatory effect of APOE on AR transcriptional activity. Furthermore, we investigated the relationship between APOE mRNA levels and the expression of classic AR target genes, such as TMPRSS2, KLK3, and NKX3.1. Consistent with our hypothesis, the mRNA levels of these AR target genes were decreased with the overexpression of APOE (**Figure 5E**). Similarly, RGX104, an LXR agonist that specifically activates APOE expression, also resulted in the downregulation of AR target gene expression in LNCaP and C4-2B parent cells (**Figure 5F**). Conversely, the knockdown of APOE in C4-2B-ENZR cells led to an increase in the transcription of AR target genes (**Figure 5G**). This regulatory effect was corroborated by immunofluorescence assays, where cells with APOE overexpression showed reduced fluorescence intensities for NKX3.1, KLK3, and TMPRSS2 in both LNCaP and C4-2B-ENZR cells (**Figure 5H-I**).

### Single cell transcriptomic landscape of Apoe-high PCa

**Figure 6:**
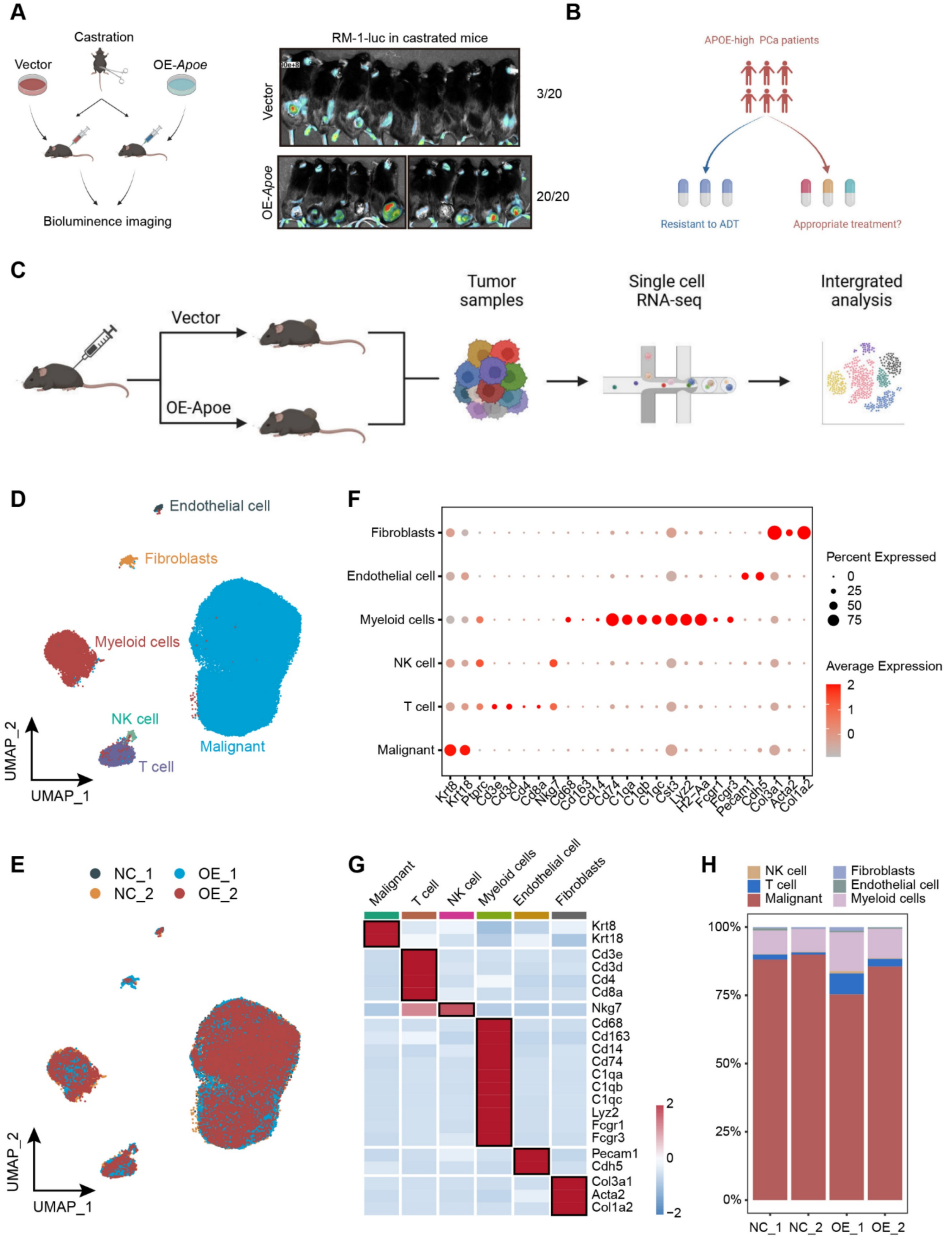
** Dissection of the molecular landscape of APOE-high PCa with scRNA-seq. (A)** The tumor formation rate was compared between castrated mice implanted with either vector control or OE-APOE RM-1 cells. **(B)** Highlighting the imperative need for tailored therapeutic strategies for the APOE-high patients. **(C)** A schematic overview of the sample processing pipeline and the scRNA-seq. **(D)** UMAP embedding illustrating the distribution of manually annotated cell types. **(E)** Sample integration was performed by “Harmony” algorithm. **(F)** Bubble plot delineating the differential expression of cell-type specific genes. Dot color intensity corresponds to gene expression levels, while dot size is indicative of the proportion of expressing cells within each cell type. **(G)** Heatmap representation of marker gene expression across distinct cell types. **(H)** Comparative analysis of cellular component alterations between the control and OE-APOE groups.

Given the demonstrated inhibitory effect of APOE on AR transcriptional activity, which contributes to the intrinsic resistance of APOE-high PCa to AR-targeted therapies, we proceeded to assess the responsiveness to ADT in APOE-high PCa. Utilizing the RM-1, an androgen-dependent mouse PCa cell line, we developed subcutaneous xenograft tumor models in C57BL/6 mice for *in vivo* validation. Following surgical castration, the overexpression of Apoe remarkably increased the occurrence of tumorigenesis, with a 100% tumor formation rate (20 out of 20), whereas the control group exhibited a tumor formation ratio of just 15% (3 out of 20) (**Figure 6A**). These results further substantiated that PCa with elevated APOE expression exhibited reduced AR dependency.

Considering the resistance to AR-targeted therapy observed in PCa patients with elevated APOE expression, identifying suitable therapeutic approaches for these patients is an imperative challenge (**Figure 6B**). In recent years, single-cell sequencing has revolutionized our comprehension of tumor heterogeneity and clonal evolution 19,20. To elucidate the biological characteristics of APOE-high PCa, we established subcutaneous tumor models for both control and APOE-overexpressing groups. We conducted single-cell RNA sequencing on four subcutaneous PCa tumors (**Figure 6C**). Utilizing stringent quality control measures, we isolated a total of 78,079 cells, comprising 65,061 malignant cells. Employing the Seurat pipeline, these cells were classified into 14 distinct clusters, which were then annotated based on their cellular identities. We identified six primary cell types: malignant cells, T cells, NK cells, myeloid cells, endothelial cells, and fibroblasts (**Figure 6D-E**), each characterized by the expression of specific marker genes (**Figure 6F-G**). Surprisingly, the analysis of cellular proportions revealed a pronounced alteration in the immune composition, especially in T cells, between the control and the Apoe overexpression group (**Figure 6H**), indicating a potential influence of APOE on the tumor microenvironment.

**Figure 7:**
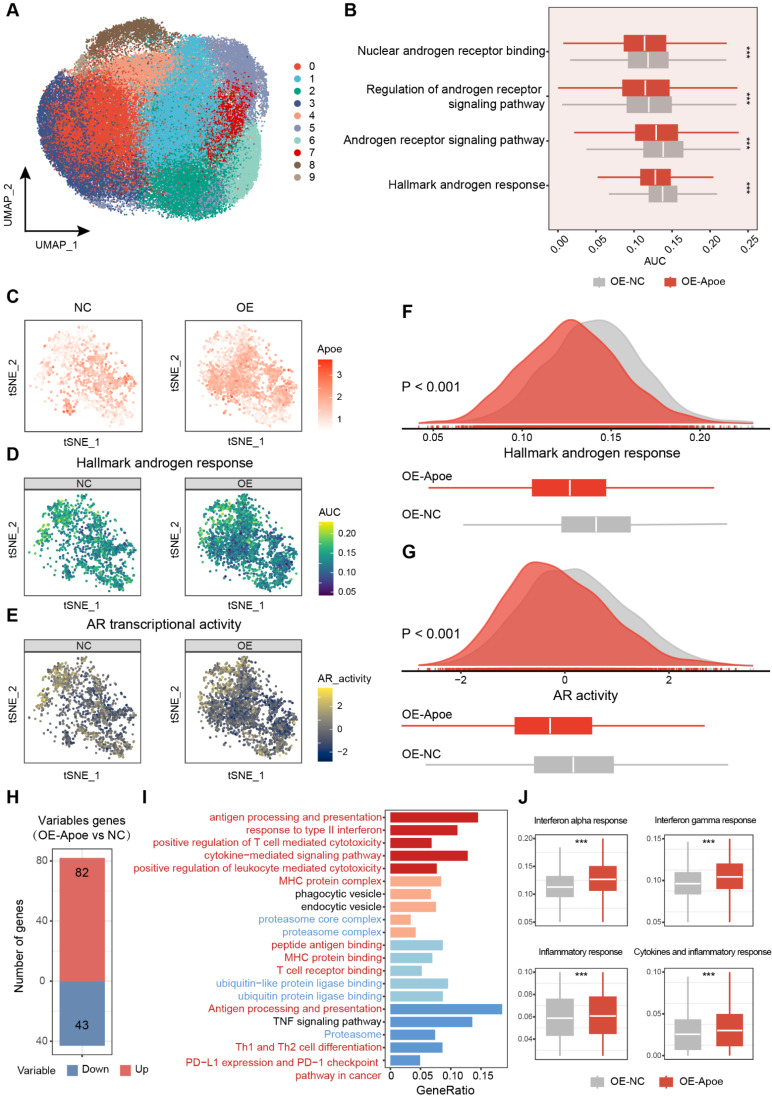
** The pivotal role of APOE in shaping the immune landscape of PCa. (A)** UMAP visualization of malignant cell clusters. **(B)** Comparative analysis of androgen response gene signatures and the AR signaling pathway between the control and APOE-overexpressing (OE-APOE) groups. **(C-G)** Assessment of the Apoe expression (C), androgen response (D), and AR transcriptional activity (E) in malignant cells with detectable APOE expression, with quantification provided in (F) and (G). **(H)** Number of differentially expressed genes in malignant cells between the control and OE-APOE groups. **(I)** Functional enrichment analysis of the differentially expressed genes in malignant cells, revealing the biological processes and pathways influenced by APOE modulation. **(J)** Comparative evaluation of immune response gene signatures between the control and OE-Apoe groups. *, p < 0.05; **, p < 0.01; ***, p < 0.001.

To further refine our analysis, we dissected the malignant cells with higher granularity. After dimension reduction and cell clustering, the malignant cells segregated into nine distinct subclusters (**Figure 7A**). By employing the "AUCell" package, a comparative analysis of androgen responsiveness was conducted on tumor cells from both the control and APOE-overexpressing groups. The results revealed a pronounced attenuation of androgen-related pathway activities in the context of elevated APOE expression (**Figure 7B**). Thereafter, we specifically examined malignant cells with detectable APOE expression to visualize the association between APOE levels and the activity of the AR signaling pathway. The results demonstrated that in PCa cells with increased APOE expression, there was a significant reduction in the activity of androgen-responsive pathways, alongside a dampened AR activity (**Figure 7C-G**). The validation was further corroborated through scRNA-seq analysis of human PCa tissues within the GSE141445 dataset, encompassing 36,424 single cells from 13 prostate tumors (**Figure S4**). We specifically isolated the malignant cells with detectable APOE expression and evaluated their androgen responsiveness and AR transcriptional activity. The results underscored that PCa cells with elevated APOE expression were coincident with a markedly attenuated activation of androgen-associated pathways and a concurrent suppression in AR activity. (**Figure S5A-C**). Additionally, a parallel pattern was observed in the relationship between TRIM25 and AR, with an inverse correlation identified between increased TRIM25 levels and the downregulation of the AR signaling pathway (**Figure S5D-F**). The results further substantiated the earlier findings, consistently supporting the concept that APOE has an inhibitory influence on the transcriptional activity of the AR.

To delve deeper into the biological characteristics of tumor cells with elevated Apoe expression, we conducted a comparative analysis to identify differentially expressed genes among malignant cells between the control and Apoe-overexpressing groups (**Figure 7H**). The functional enrichment analysis uncovered a robust correlation between Apoe and the ubiquitin-proteasome system, echoing our earlier observations (**Figure 7I**). Apart from this, we observed a significant link between Apoe and the immune response in PCa (**Figure 7I**). Signaling pathways pertinent to the immune response, such as the Interferon gamma response, Interferon alpha response, Inflammatory response, and Cytokines and inflammatory response pathways, were markedly upregulated in the Apoe-high group (**Figure 7J**). This association was further corroborated in the GSE141445 dataset (**Figure S5G**), underscoring the potential immunomodulatory role of Apoe in the tumor microenvironment. Previous studies have primarily concentrated on the role of APOE in specific immune cells and its impact on the tumor microenvironment, specifically in relation to myeloid-derived suppressor cells (MDSCs) and macrophages 16,21,22. Hence, it is necessary to explore the impact of APOE on immune responses and the effectiveness of immunotherapy in PCa.

### APOE overexpression sensitizes PCa cells to ICI therapy

**Figure 8:**
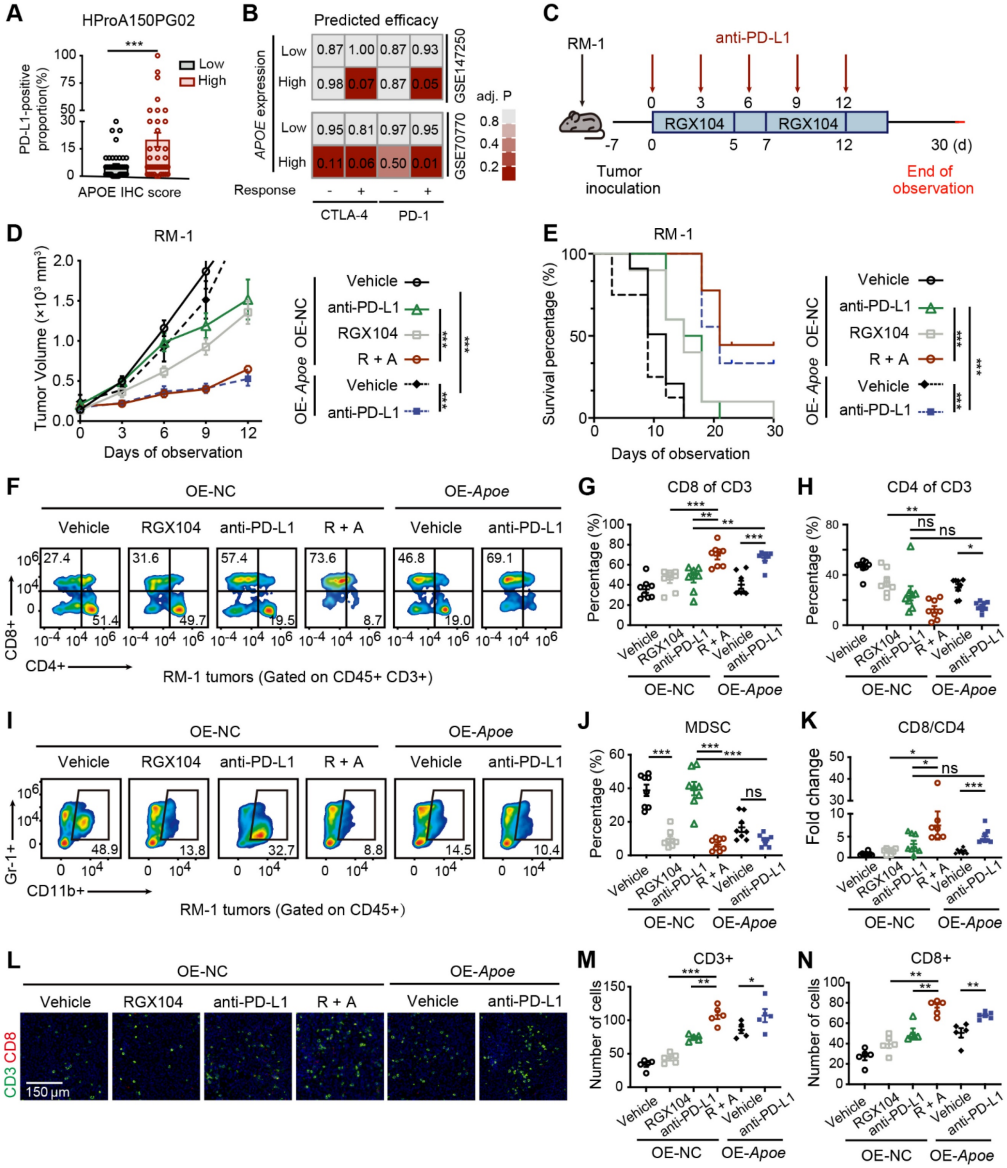
** Overexpression of APOE enhances the sensitivity of PCa cells to ICI. (A)** The proportion of PD-L1-positive tumor area in APOE-high and APOE-low patients from the PCa tissue assay (HProA150PG02) was compared. **(B)** Using an anti-PD-1/CTLA4-treated melanoma immunotherapy cohort as a reference, subclass mapping was performed on two datasets featuring CRPC (GSE70770 and GSE147250). High APOE expression was significantly linked to patient response to anti-PD-1/CTLA4 treatment. **(C)** A flowchart depicting the administration of the combination of RGX-104 and anti-PD-L1 therapy in animal models is provided. **(D-E)** Tumor volume progression of RM-1 tumors overexpressing Apoe or control treated with vehicle, anti-PD-L1, RGX104, or combination therapies plotted over time (D). Survival analysis of mice subjected to the aforementioned treatments (E). **(F-H, K)** Representative flow cytometry images (F) and corresponding quantitative analysis (G-H, K) of TILs in the indicated treatment groups. **(I, J)** Representative flow cytometry images (I) and quantitative analysis (J) of MDSCs in the specified experimental groups. **(L-N)** Representative immunofluorescence images (L) and corresponding quantitative analysis (M, N) of TILs in the indicated treatment groups. *, p < 0.05; **, p < 0.01; ***, p < 0.001. Abbreviations: TIL, tumor-infiltrating lymphocyte; MDSC, myeloid-derived suppressor cells.

Correlation analyses across multiple PCa cohorts uncovered a robust co-expression pattern of APOE alongside immune markers, including the immune checkpoints CD274 (PD-L1) and PDCD1LG2 (PD-L2), alongside various chemokines and antigen-presenting surface markers (**Figure S6A**). Further investigation of the relationship between APOE and predicted immune checkpoint inhibitor (ICI) response signatures disclosed a positive correlation between APOE and the enrichment scores associated with these immunotherapy-related signatures (**Figure S6B**). Moreover, IHC staining of the PCa tissue microarray indicated a higher proportion of PD-L1 positivity in tumors with high APOE expression (**Figure 8A**). These findings pointed to a significant reshaping of the tumor microenvironment in APOE-high tumors, suggesting a potential therapeutic advantage in employing ICIs for treatment. Using the ICBatlas database, we conducted a comprehensive analysis that included diverse cancer type that had received anti-PD1 therapy, including melanoma, non-small cell lung cancer, glioblastoma, renal cell carcinoma, and gastric cancer. Survival analysis indicated that patients with high APOE expression had a significantly longer PFS compared to the low expression group (**Figure S6C**). Furthermore, we engaged in subclass mapping analysis to anticipate the link between APOE expression levels and the responsiveness to immunotherapy in PCa. Strikingly, tumors with high APOE expression were projected to exhibit a robust response to PD-1 and CTLA-4 inhibitory agents within two cohorts of CRPC (**Figure 8B**). This insight revealed a promising therapeutic opportunity for the utilization of ICIs in tumors with elevated APOE levels.

To further assess the impact of APOE induction on the efficacy of immunotherapy, RM-1 cells were utilized to establish a xenograft tumor model to evaluate the response to anti-PD-L1 treatment under conditions of APOE induction, induced either by RGX104 treatment or Apoe overexpression (**Figure 8C**). Notably, robust suppression of tumor growth was observed in OE-NC tumors following treatment with RGX104 in combination with anti-PD-L1, as well as in tumors overexpressing Apoe treated with anti-PD-L1, when compared to OE-NC tumors treated with anti-PD-L1 alone (**Figure 8D**). Concurrently, a significantly prolonged survival rate was observed in mice bearing tumors treated with OE-NC + RGX104 + anti-PD-L1 and OE-APOE + anti-PD-L1, as opposed to OE-NC tumors with anti-PD-L1 alone (**Figure 8E**). In our subsequent investigation, we examined the tumor microenvironment after anti-PD-L1 treatment. The combination of RGX104 with anti-PD-L1 or Apoe-OE with anti-PD-L1 markedly enhanced CD8+ T-cell infiltration and elevated the CD8/CD4 proportion ratio within the tumors, without affecting the draining lymph nodes or spleens (**Figure 8F-H, K and Figure 7A-B**), which indicating a potentiated antitumor immune response in APOE-high tumors treated with anti-PD-L1. Besides this, staining for CD4 and CD8 further confirmed the increased immune cell infiltration following APOE induction and anti-PD-L1 treatment (**Figure 8L-N**). Prior studies have indicated that RGX104 could disrupt MDSC survival, thereby triggering an antitumor innate immune response 21. In alignment with these findings, we observed a reduction in MDSC abundance in mice treated with RGX104 or RGX104 in combination with anti-PD-L1, both within the tumors and in the spleens (**Figure 8I-J and Figure S7C**). Interestingly, Apoe-OE also curtailed MDSC infiltration into the tumors (**Figure 8I-J**). These findings suggested that APOE, whether induced systemically via an agonist or intrinsically within the tumor, could exert a suppressive effect on MDSCs. APOE holds the potential to revitalize the tumor microenvironment and augment the efficacy of ICI therapy. Consequently, despite resistance to AR-targeted therapy, APOE-high PCa may benefit from ICI treatment.

## Discussion

Endocrine therapy, designed to impede the AR signaling pathway, stands as the primary treatment modality for advanced PCa 23. However, resistance to AR-targeted therapy is a significant determinant of patient prognosis, often leading to therapeutic challenges 24,25. Despite advancements in the development of novel agents that target the AR signaling axis, both primary and acquired resistance to these treatments are common occurrences 17. In recent years, the advent of precision medicine has heightened the need for more refined methods of selecting suitable therapeutic strategies. Traditional clinical indicators, including PSA levels, ISUP grading, and TNM staging, have shown limitations due to the heterogeneity of tumors. Consequently, the identification and selection of appropriate biomarkers have significant importance in guiding clinical decisions pertaining to tumor treatment 26. This study conducted a comprehensive analysis of seven PCa cohorts to identify candidate genes that play a crucial role in disease progression and resistance to AR-targeted therapy. The findings revealed significantly increased expression of APOE in enzalutamide-resistant PCa cell lines and CRPC. A pronounced correlation was identified between heightened APOE expression and adverse prognostic indicators, along with clinicopathological features. Substantiating these findings, both *in vivo* and *in vitro* studies confirmed that APOE overexpression significantly contributes to AR-targeted therapy resistance in PCa. Additionally, this investigation proved the potential of APOE as a prognostic biomarker for predicting the responsiveness of PCa patients to anti-androgenic drugs within our TJ cohort.

Numerous studies have demonstrated a strong correlation between the activity of the AR signaling pathway and resistance to AR targeted therapy 27-29. In light of this, we delved deeper into the potential interplay between APOE and AR, and the findings of this study revealed that APOE exerted regulatory control over AR protein expression. Multiple studies have documented that the AR undergoes post-transcriptional modifications such as phosphorylation, acetylation, SUMOylation, methylation, and ubiquitination, which have significant roles in regulating the structure, activity, and stability of the AR 30-32. Among them, ubiquitination is a versatile post-translational modification mechanism for AR, which involves ubiquitin modification of substrates and sequential degradation by the proteasome 33-35. TRIM25, a member of the TRIM protein family, is increasingly recognized for its significant impact on a variety of physiological conditions, including innate immunity and cancer 36-38. TRIM25 has been reported to target key regulatory proteins such as p53, PTEN, and Keap1 for degradation, thereby exerting a fundamental influence on the control of cancer cell proliferation, metastasis, and the mediation of chemotherapy resistance 37,39,40. In this study, we presented evidence illustrating the interaction between APOE and AR, which facilitated the ubiquitination and subsequent degradation of AR by enhancing the interaction between TRIM25 and AR. In addition to our previous investigations, we conducted studies to evaluate the influence of APOE on the transcriptional activity of the AR. Utilizing an AR response reporter, we directly assessed the transcriptional activity of AR in PCa. Our results demonstrated that the overexpression of APOE significantly dampened the transcriptional activity of AR. On the single-cell level, a pronounced correlation was identified between increased APOE expression and reduced androgen sensitivity, coupled with diminished AR activity. Furthermore, data derived from Co-IP assays suggest that APOE exhibited binding affinity for the NTD of AR, playing a pivotal role in the modulation of its transcriptional activity. Collectively, APOE appeared to exert a dual effect in PCa by promoting the degradation of AR and simultaneously hindering its transcriptional activity. As a result, the attenuated AR activity in APOE-high PCa contributed to the primary resistance to AR-targeted therapy.

Given the resistance to AR-targeted therapies in patients with APOE-high PCa, there is a pressing need to investigate alternative therapeutic approaches. Recently, the advent of single-cell sequencing has significantly advanced our understanding of tumor heterogeneity. In this context, we undertook single-cell RNA sequencing to discern the biological characteristics of tumor cells with elevated Apoe expression. The functional enrichment analysis has unveiled a substantial correlation between Apoe and immune response, highlighting the potential for Apoe to modulate the tumor microenvironment. Furthermore, our tissue array data indicated a significantly higher prevalence of PD-L1 positivity in the group with elevated APOE expression. This prompted our hypothesis that APOE might serve as a predictive biomarker for immunotherapy responsiveness in PCa.

In the last decade, there has been a marked increase in clinical trials exploring immunotherapy for various solid tumors 41,42. With recent advancements in understanding immune mechanisms and the advent of sophisticated molecular diagnostics, immunotherapy is gaining traction as a treatment option for PCa 43. To further substantiate the influence of APOE on the modulation of the tumor microenvironment, we conducted *in vivo* animal experiments. The outcomes demonstrated significant tumor growth inhibition in the OE-NC group subjected to the combined treatment of RGX104 and anti-PD-L1, as well as in the OE-Apoe RM-1 group treated with anti-PD-L1, contrasting with the OE-NC group treated with anti-PD-L1 alone. Previous research have delineated the regulatory role of the LXR/ApoE axis in innate immune suppression, where LXR agonism, specifically RGX-104, has been observed to diminish the presence of MDSCs in both mice and patients 21. Our research corroborated these findings, with a noted reduction in MDSC abundance and a concomitant increase in CD8+ T-cell infiltration within the OE-Apoe group. These observations suggested that immunotherapy could present a viable therapeutic strategy for PCa with elevated APOE expression.

Consequently, given the considerable heterogeneity observed among individuals with PCa, we propose for the use of pathological immunohistochemistry results of APOE to guide adjuvant therapeutic strategies for patients with high-risk PCa. Patients with elevated APOE expression may have relatively diminished activity within the AR signaling pathway, implying that their clinical response to AR-targeted therapies might be constrained. Therefore, alternative adjuvant interventions should be contemplated. For advanced PCa patients with high APOE expression, immunotherapy could represent a potent treatment alternative.

Certainly, several limitations in this study also need to be acknowledged. The number of patients included in our own PCa treatment cohort is comparatively limited, and a prospective cohort study is needed to further validate the impact of APOE on primary resistance to AR-targeted therapy in PCa patients. Additionally, while our findings suggest that tumor-intrinsic APOE plays a role in shaping the tumor microenvironment, the precise mechanism through which APOE regulates PD-L1 expression and influences immune modulation requires further investigation. Moreover, to better establish the translational relevance of APOE in PCa, future studies should incorporate additional preclinical models, such as patient-derived xenografts and organoid models, to further validate its therapeutic potential.

In conclusion, our study supports the use of APOE as a promising biomarker for enhancing therapeutic decision-making in PCa. Immunotherapy, rather than AR targeted therapy, is more appropriate for PCa patients with high levels of APOE. Additionally, targeting APOE may enhance the therapeutic efficacy of anti-androgenic treatment and ICI therapy.

## Materials and methods

### Patients

Our study encompassed a cohort of 57 Chinese patients, all diagnosed with localized PCa. They underwent radical prostatectomy at Tongji Hospital within the timeframe of January 2017 to August 2019. The patients selected adhered to the following criteria: 1) age ≥ 18 years; 2) histologically confirmed prostate adenocarcinoma; and 3) categorized as either high-risk PCa (characterized by Gleason scores ≥ 8 or preoperative serum PSA ≥ 20 ng/mL) or locally advanced PCa (assessed as pT3/pT4, N0M0, or any T, N1M0). Post-surgery, all patients were subjected to a treatment regimen combining ADT with anti-androgenic therapy. Retrospective assessments were made up to August 2023 to monitor patient recurrence patterns. **Table 1** provides an in-depth account of the patient specifics. Ethics approval from the Tongji Hospital Ethics Committee was acquired prior to this investigation. Moreover, informed consent was also procured from all participating patients concerning the use of tissue samples for scientific research.

### Cell lines and reagents

The LNCaP, C4-2, C4-2B, PC3, DU145, 22RV1, VCaP and HEK293T cell lines were purchased from the American Type Culture Collection (ATCC, Manassas, USA). These cell lines were cultivated in accordance with the instructions supplied by ATCC. The C4-2B-ENZR was established by exposing C4-2B cells to an initial dose of enzalutamide (5 µM) and gradually increasing concentrations up to 10 µM for 16 weeks, followed by culturing with media containing 10 µM enzalutamide (**Figure S2A**). Furthermore, CCK-8 and colony formation assays were performed to validate the established resistance to enzalutamide (**Figure S2B-C**). All cell lines were cultured in a clean incubator at 37 °C and 5% CO_2_.

Cycloheximide, the proteasome inhibitor MG132, RGX-104 and enzalutamide were all purchased from Selleck Chemicals (Houston, USA). Cycloheximide was added and remained until harvest within the medium and at a final concentration of 10 µg/ml. At the indicated time points after CHX introduction, the cells were lysed, and total protein was harvested for subsequent immunoblot analysis. After treatment with 10 µM MG132 for 24 h, total protein was extracted to evaluate the functions of the ubiquitin-proteasome system in APOE-mediated AR degradation.

### Plasmids and cell transfection

Viral particles with short hairpin RNA (shRNA) targeting APOE (sh-*APOE*) and non-target shRNA were designed and synthesized by Viraltherapy Technologies (Wuhan, China). The RNAi sequences were as follows: sh-1#: 5′-GCAGGAAGATGAAGGTTCTGT-3′; sh-2#: 5′-GAAGGAGTTGAAGGCCTACAA-3′; sh-3#: 5′-GCAGACACTGTCTGAGCAGGT-3′. Viral particle transfection was performed by adding polybrene to the culture medium, and cells were screened with puromycin (10 μg/mL). The knockdown efficiency was evaluated by quantitative real-time PCR (RT-qPCR) and immunoblot analysis. Vector expressing Flag-APOE was also acquired from Viraltherapy technologies. The AR response reporter, vectors expressing Flag-TRIM25, HA-AR-full length and all truncated mutants of AR were obtained from Miaoling Biotechnology. Transfection of plasmids was conducted by using Lipo3000 (Thermo Fisher Scientific, USA) following protocols provided by the manufacturer.

### RT-qPCR, co-immunoprecipitation and immunoblotting

Extraction of total cellular RNA was performed using FastPure Cell/Tissue Total RNA Isolation Kit V2 (Vazyme, Nanjing, China), and reverse transcription was performed using PrimeScript RT Master Mix. The qRT-PCR process was completed according to a previous description 44. GAPDH served as an endogenous control, and the corresponding primer sequences are listed in **Table S8**. Furthermore, cells were lysed using RIPA lysis buffer and augmented with PMSF (Servicebio Technology, Wuhan, China) and phosphatase inhibitor cocktail (Boster Biological Technology, Wuhan, China) to procure total protein. For the co-IP assay, protein lysate was prepared with NP40 buffer coupled with PMSF and cocktail phosphatase inhibitor. Both co-IP and subsequent immunoblotting were executed following a previously established protocol 45. More information regarding the primary and secondary antibodies used for this study can be found in **Table S9**.

### LC-MS/MS mass spectrometry

LNCaP cells were transfected with a Flag-APOE vector, after which the cells were lysed and the total protein was collected. Immunoprecipitation assays were conducted using anti-Flag or anti-AR antibodies for further analysis by mass spectrometry. The LC-MS/MS data collection was conducted using an AQ Executive Plus mass spectrometer linked with an EASY-nLC 1200 system, following the methods provided by the manufacturer. The processing of raw data was performed using the MaxQuant program using the Andromeda database search method. The spectra files were searched using the UniProt human proteome database to identify the binding proteins of APOE and AR, with a false discovery rate of 1% applied at both the peptide and protein levels.

### Cell viability and colony formation assays

The cells were seeded onto 96-well plates at a density of 2000 cells per well. The assessment of cell viability was conducted in accordance with the Cell Counting Kit-8 procedure (40203ES60, Yeasen Biotechnology, Shanghai, China). For the colony formation assay, cells were inoculated onto a 6-well plate at a density of 3000 cells per well and then exposed to either enzalutamide (40 µM) or DMSO. After 14 days of incubation, the colonies were harvested and enumerated for statistical analysis.

### IHC staining and scoring

Two PCa tissue arrays (HProA120Su01 and HProA150PG02) were acquired from Shanghai Outdo Biotech Company (Shanghai, China). The HProA120Su01 array is composed of a sample pool from 60 PCa patients and simultaneously features their corresponding survival information (**Table S10**). In contrast, while the HProA150PG02 array lacks specific patient survival data, it encompasses the proportion of PD-L1-positive areas in each patient (**Table S11**). The tissue samples from Tongji Hospital were fixed in paraffin, sliced into sections, and then subjected to immunohistochemical staining. IHC staining was performed following the guidelines provided by the manufacturer of the IHC kit (Yeasen Biotechnology, Shanghai, China). The protein quantification for each sample was assessed by a pathologist blinded to the clinical information. The modified H-score, which incorporates the percentages of mild (1+), moderate (2+), and strong staining (3+), was used to indicate the amount of protein expression. This scoring system ranges from 0 to 300. Samples with too few tumor cells (<300 cells per case) were excluded and not used for further analysis.

### Immunofluorescence microscopy

Immunofluorescence was conducted according to an established protocol 45. Briefly, cells were cultured on coverslips placed in a 24-well plate. After 24 h, the cells were washed with PBS, fixed with 1% paraformaldehyde for 20 mins, permeabilized with 0.5% Triton for 30 mins and blocked with BSA for 1 h. Subsequently, the cells were incubated with primary antibodies at 4 °C overnight, followed by washing with PBS and incubation with secondary antibodies for 1 h the next day. DAPI staining (Solarbio, Beijing, China) for 5 mins was then performed, and coverslips were then mounted for subsequent microscopy. The primary and secondary antibodies are listed in **Table S9**.

### Animal studies

All *in vivo* experiments obtained approval from the Institutional Animal Care and Use Committee of Huazhong University of Science and Technology (TJH-202110018). Both BALB/c nude and C57BL/6 male mice were procured from Shulaibao Biotechnology (Wuhan, China) and were raised in specific pathogen free (SPF) conditions. For the establishment of animal tumor models, all APOE-overexpressing or APOE-depleted cell lines were generated via lentiviral transduction. To investigate the role of APOE in mediating enzalutamide resistance, a total of 5.0×10^6^ C4-2B-ENZR cells (or 8.0×10^6^ C4-2B-Parental cells) expressing shNC (or OE-NC) or shAPOE (or OE-APOE) were mixed with Matrigel (following a 1:1 ratio) and injected subcutaneously into mice. Mice were divided into four groups: (1) shNC (or OE-NC) + DMSO, (2) shAPOE (or OE-APOE) + DMSO, (3) shNC (or OE-NC) + enzalutamide (20 mg/kg/d, p.o.), and (4) shAPOE (or OE-APOE) + enzalutamide (20 mg/kg/d, p.o.). The subcutaneous tumors were measured by a caliper every 4 days, and tumor volume was calculated by V = [length × width^2^] × 0.5. After 28 days of treatment, the mice were sacrificed, and the tumors were removed for analysis. To investigate the role of APOE in the immunotherapy response, 1 × 10^6^ OE-NC or OE-Apoe RM-1 cells were subcutaneously injected into the lower flank of C57BL/6 mice. Mice were randomly assigned into each treatment group (n=8) seven days after tumor transplantation and received vehicle, RGX-104, anti-PD-L1 or combinations. RGX-104 was administered intraperitoneally (40 mg/kg/day) consecutively for 5 days with a 2-day break. Anti-PD-L1 was administered (10 mg/kg) every three days. Tumor volume was evaluated every three days. Mice were euthanized by CO_2_ inhalation when the tumor volume reached 2000 mm^3^ as a humane endpoint.

### Flow cytometry analysis

To measure the apoptosis rate, cells were harvested and double stained with Annexin V-PE and 7-AAD (Yeasen Biotechnology, Shanghai, China). This was followed by an incubation period of 10 to 15 mins in a dark setting at room temperature. The data collection for flow cytometry was conducted using a CytoFlex cytometer (Beckman Coulter, USA). For tumor microenvironment evaluation. Subcutaneous tumors were harvested 7 days after initial therapy, and a tumor dissociation kit (130-096-730; Miltenyi Biotec, San Diego, CA, USA) and gentle MACS Dissociator (Miltenyi Biotec) were used to prepare single-cell suspensions, followed by red blood cell (RBC) lysis and live/dead cell staining using a Zombie Aqua Fixable Viability Kit (BioLegend, San Diego, CA, USA). Subsequently, blocking of CD16/32 and cell marker staining was performed. After thorough washing, the cells were resuspended in PBS and analyzed using a CytoFlex cytometer. The gating strategy for T cells and MDSCs is shown in **Figure S8**.

### Single cell RNA-seq analysis

Single-cell libraries derived from RM-1 tumor of mice treated with vehicle or OE-Apoe were generated utilizing the 10X Genomics Chromium Controller Instrument and Chromium Single Cell 3' V3 Reagent Kits (10X Genomics, Pleasanton, CA) according to the manufacturer's guidelines. The sequencing was executed on the Illumina NovaSeq platform, following the protocol provided by the manufacturer. The experimental procedures were carried out by Novogene Co., Ltd. Comprehensive methodologies concerning the processing of scRNA-seq data are delineated in the **Supplementary material**.

### Bioinformatics analysis

The GSE116918, GSE46602, GSE32269, GSE151083, GSE150807, GSE70770, GSE147250 and GSE94767 datasets were downloaded from the Gene Expression Omnibus (GEO). The transcriptional profile and clinical details of the PRAD cohort were obtained from The Cancer Genome Atlas (TCGA), and the DKFZ, MSKCC and Su2c cohorts were acquired via the cBioPortal database (https://www.cbioportal.org/). The correlation between APOE expression and the effectiveness of immunotherapy was assessed by ICBatlas 46 (http://bioinfo.life.hust.edu.cn/ICBatlas/). Differentially expressed genes were screened by utilizing “limma” for microarray data and “DEseq2” for RNA-seq data (fold change > 2, p < 0.05). Univariate Cox analysis was utilized to identify prognostic genes (p < 0.01). The lists of the genesets reflecting the androgen response and AR activity are illustrated in **Table S12**, and immunotherapy-predicted pathways were collected from a previous study 47. The “ssGSEA” algorithm was used to evaluate the enrichment score in each sample. Drug sensitivity analysis was accomplished by utilizing transcription data and IC50 or AUC values of anti-androgenic drugs from the CPG and PRISM databases by using the R package "pRRophetic" 48. Prediction of APOE expression in affecting immunotherapy response was performed by using subclass mapping to compare the similarity of gene expression profiles between the PCa cohort and an immunotherapy cohort of melanoma accepting anti-CTLA4/PD-1 49,50.

### Statistical analysis

The data are presented as the means ± standard deviations. The statistical tests used for comparing two groups were Student's t test for normally distributed data and the Wilcoxon test for skewed distribution data. One-way ANOVA followed by Tukey's multiple comparison test was used to compare multiple groups. Spearman correlation was used to conduct all correlation analyses. The log-rank test was used to assess the survival curves. Statistical analyses were conducted using R version 4.3.0 and GraphPad Prism version 8. Significance was determined at a threshold of p < 0.05.

## Supplementary Material

Supplementary figures and methods.

Supplementary tables.

## Figures and Tables

**Table 1:** Clinical information of Tongji PCa cohort

Clinicopathological features	No. of patients (N=57)
**Age**	
≤70	38 (66.7%)
>70	19 (33.3%)
**Gleason score**	
<8	27 (47.4%)
≥8	30 (52.6%)
**Preoperative TPSA**	
≤20	24 (42.1%)
>20	33 (57.9%)
**Pathological T stage**	
pT2	13 (22.8%)
pT3	25 (43.9%)
pT4	19 (33.3%)
**Pathological N stage**	
N0	39 (68.4%)
N1	18 (31.6%)
**Progression free survival**	
No	47 (82.5%)
Yes	10 (17.5%)

## Abbreviations

ADT: Androgen deprivation therapy
AR: Androgen receptor
CHX: Cycloheximide
Co-IP: Co-immunoprecipitation
CRPC: Castration-resistant prostate cancer
DBD: DNA binding domain
ICI: Immune checkpoint inhibitor
LBD: Ligand binding domain
MDSC: Myeloid-derived suppressor cell
NTD: N-terminal domain
PCa: Prostate cancer
TRIM25: Tripartite motif 25
