# Supplementary material for: Apolipoprotein E promotes primary resistance to AR-targeted therapy via inducing TRIM25-mediated AR ubiquitination and sensitizes immunotherapy in prostate cancer
Source: Theranostics. 2025 Apr 21;15(12):5572–91. doi: 10.7150/thno.109994 (PMC12068304; doi:10.7150/thno.109994)
Supplement: Supplementary file 1 — Supplementary figures and methods. [file thnov15p5572s1.pdf]

Supplementary Figures

Apolipoprotein E promotes primary resistance to AR-targeted therapy via inducing TRIM25-mediated AR ubiquitination and sensitizes immunotherapy in prostate cancer

Chaofan Liu[1, †], Xi Wang[2, †], Qinyu Li[1, †], Xintao Gao[3], Kai Zeng[2], Beining Li[2], Jianping Miao[4], Bolong Zheng[5], Jihong Liu[2], Zhihua Wang[2, *], Xianglin Yuan[1, *], Bo Liu[1, *]

[1]Department of Oncology, Tongji Hospital, Tongji Medical College, Huazhong University of Science and Technology, Wuhan 430030, Hubei, China
[2]Department of Urology, Tongji Hospital, Tongji Medical College, Huazhong University of Science and Technology, Wuhan 430030, Hubei, China
[3]Department of Urology, Sir RunRun Shaw Hospital, College of Medicine, Zhejiang University, Hangzhou, China.
[4]Department of Geriatrics, Tongji Hospital, Tongji Medical College, Huazhong University of Science and Technology, Wuhan, Hubei, China
[5]School of Computer Science and Technology, Huazhong University of Science and Technology

† These authors contributed equally to this work and shared first authorship.

Corresponding to:

Professor Bo Liu, boliu888@hotmail.com

Professor Xianglin Yuan, yuanxianglin@hust.edu.cn

Professor Zhihua Wang, zhwang_hust@hotmail.com

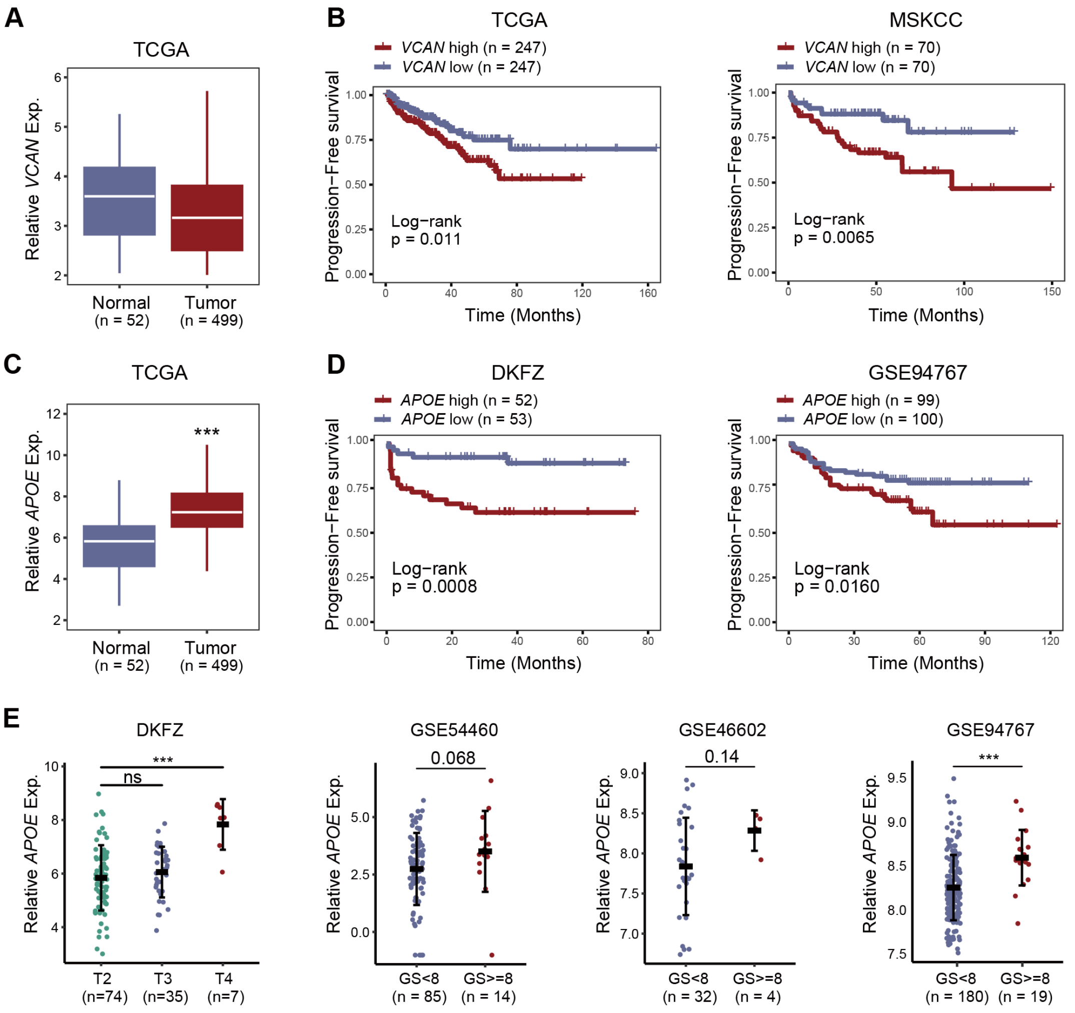

Figure S1: Correlation between VCAN and APOE expression and PCa patient prognosis.

(A) Comparison of VCAN gene expression in PCa tissue and normal prostate tissue.

(B) To assess the correlation between the PFS of patients and the expression of the VCAN gene in PCa, Kaplan-Meier analysis was undertaken using the TCGA and MSKCC databases.

(C) Comparison of APOE gene expression in PCa tissues and normal prostate tissues.

(D) Using the DKFZ and GSE94767 datasets, Kaplan-Meier analysis was carried out to determine the association of PFS of PCa patients with the expression of APOE.

(E) A significant positive correlation was observed between higher APOE expression and increased pathological T stage and a higher Gleason score. ***, $p < 0.001$.

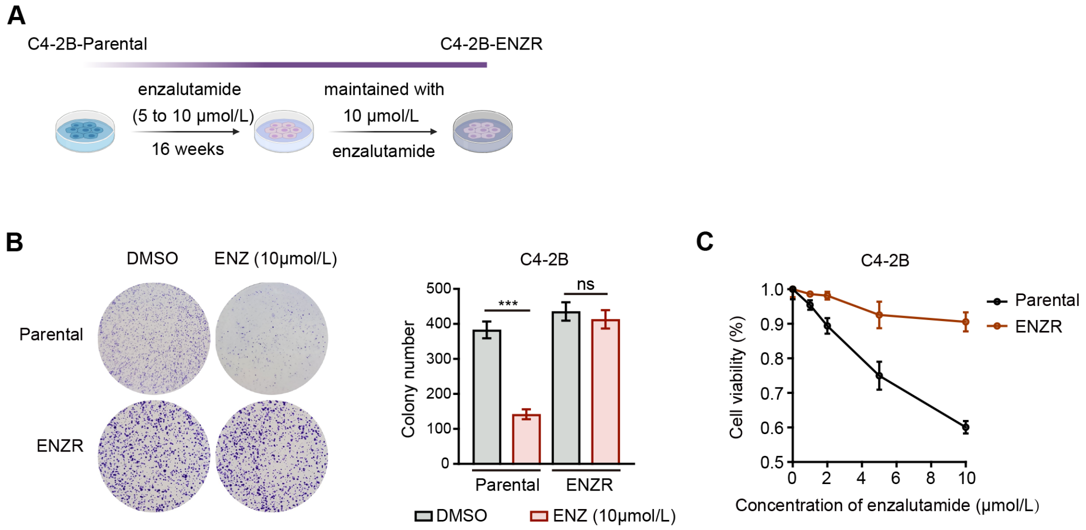

Figure S2: Establishment of the C4-2B ENZ-R cell line.

(A) Schematic drawing showing the establishment of the enzalutamide resistant C4-2B cell line (C4-2B-ENZR).

(B-C) The resistance of the C4-2B-ENZR cell line was confirmed using colony formation (B) and CCK8 (C) assays.

***, $p < 0.001$.

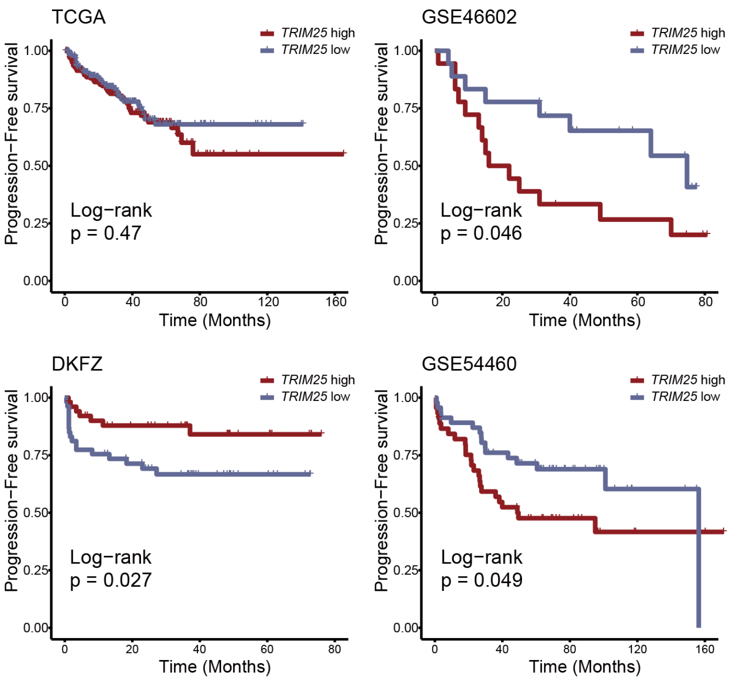

Figure S3: Correlation between TRIM25 gene expression and PCa patient prognosis. Kaplan-Meier survival analysis was conducted using data from the TCGA, GSE46602, GSE70770, DKFZ, and MSKCC datasets to evaluate the relationship between PFS of PCa patients and the expression levels of the TRIM25 gene.

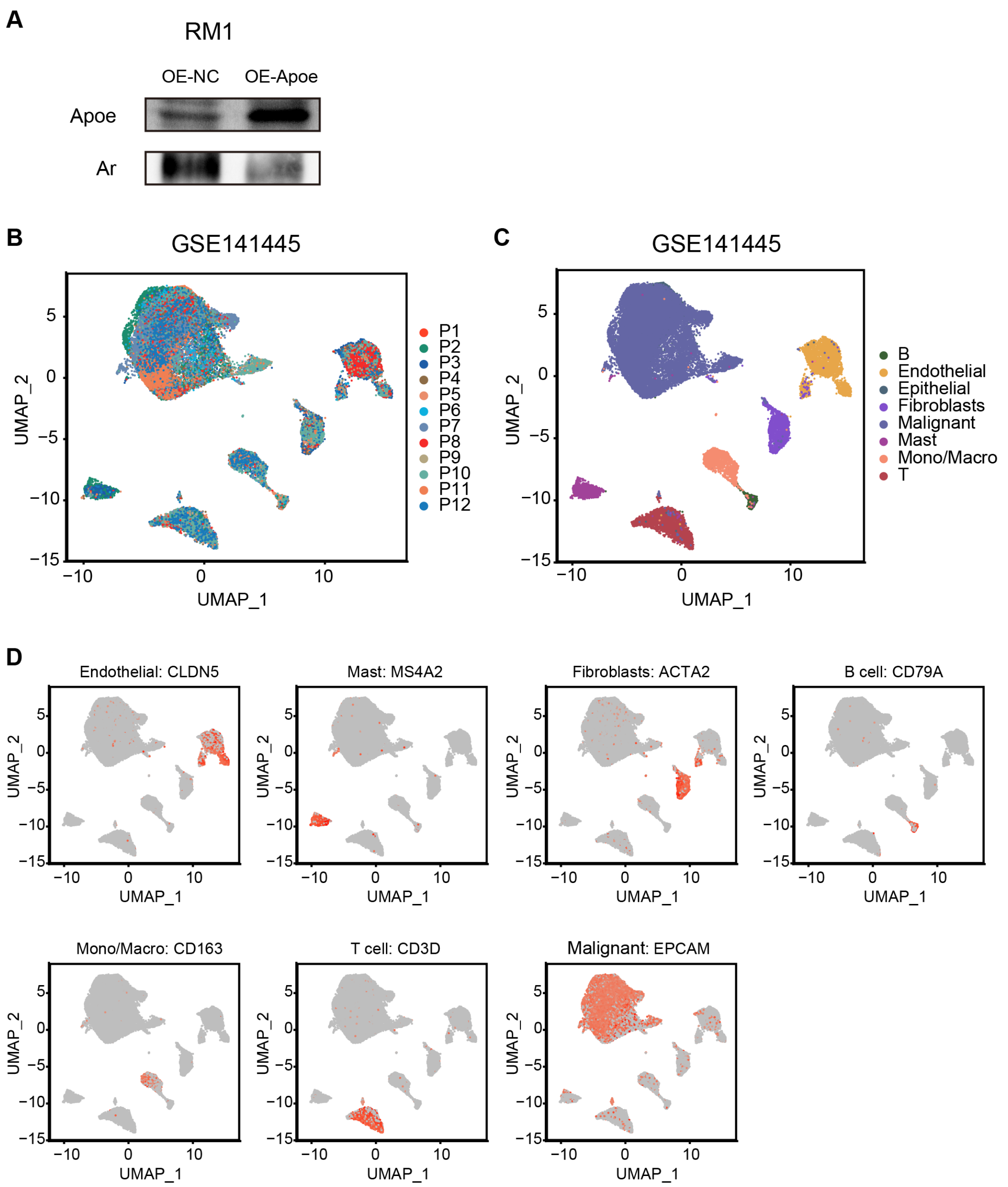

Figure S4: Dissection of the molecular landscape of APOE-high PCa with scRNA-seq.

(A) Validation of Apoe and Ar expression in the RM-1 cells.

(B-C) UMAP plot showing the distribution of the cluster (B) and cell types (C).

(D) UMAP plot showing the subclusters of endothelial, mast, fibroblasts, B cell, monocyte/macrophage, T cell and malignant cell.

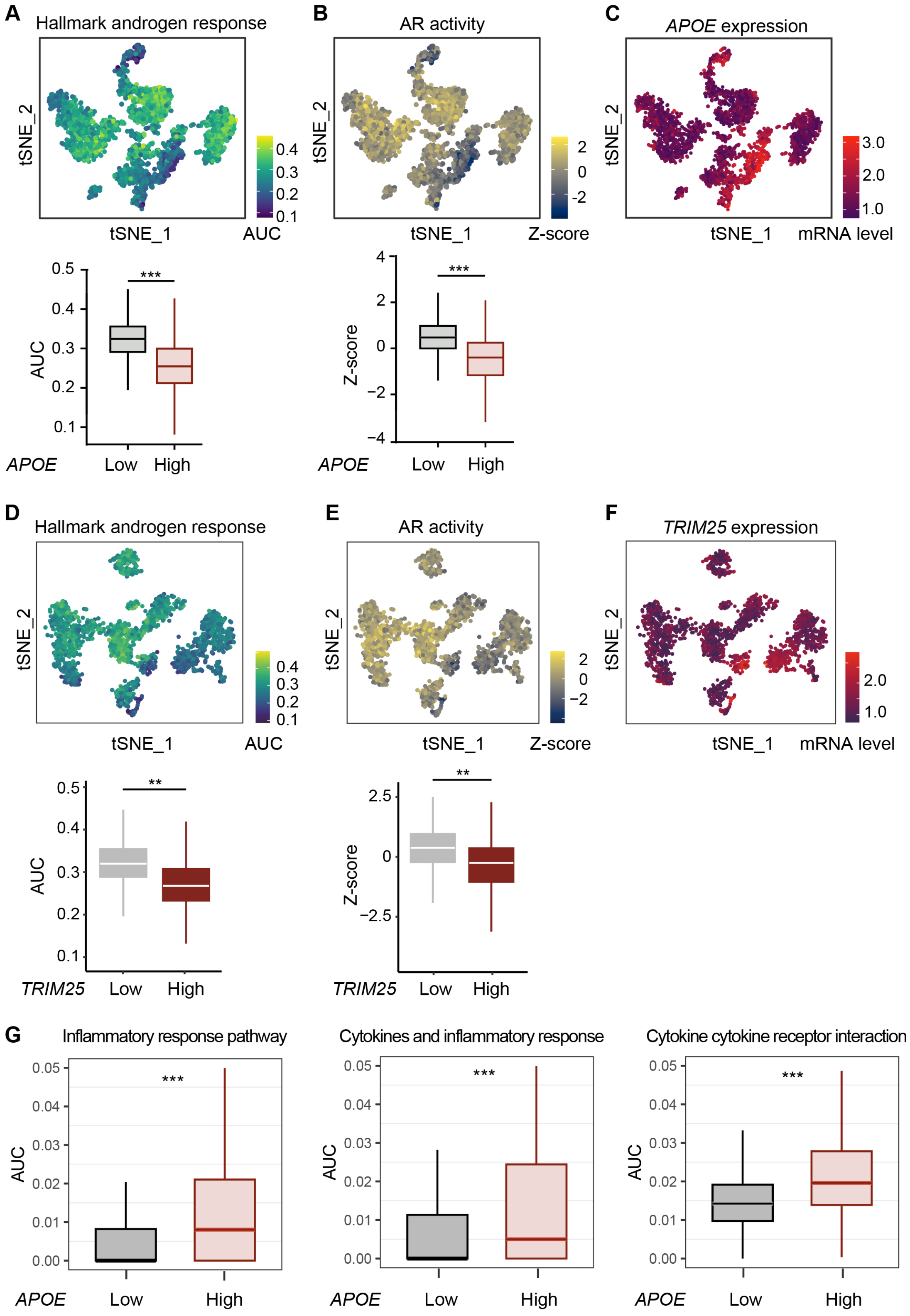

Figure S5: AR signaling activity was suppressed by APOE and TRIM25

(A-C) Comparisons were made between groups with high and low APOE gene expression in terms of androgen response gene signatures and AR signaling pathway activities.

(D-F) Comparisons were made between groups with high and low TRIM25 gene expression in terms of androgen response gene signatures and AR signaling pathway activities.

(G) Comparing the immune response gene signatures between groups with high and low APOE gene expression.

***, $p < 0.001$.

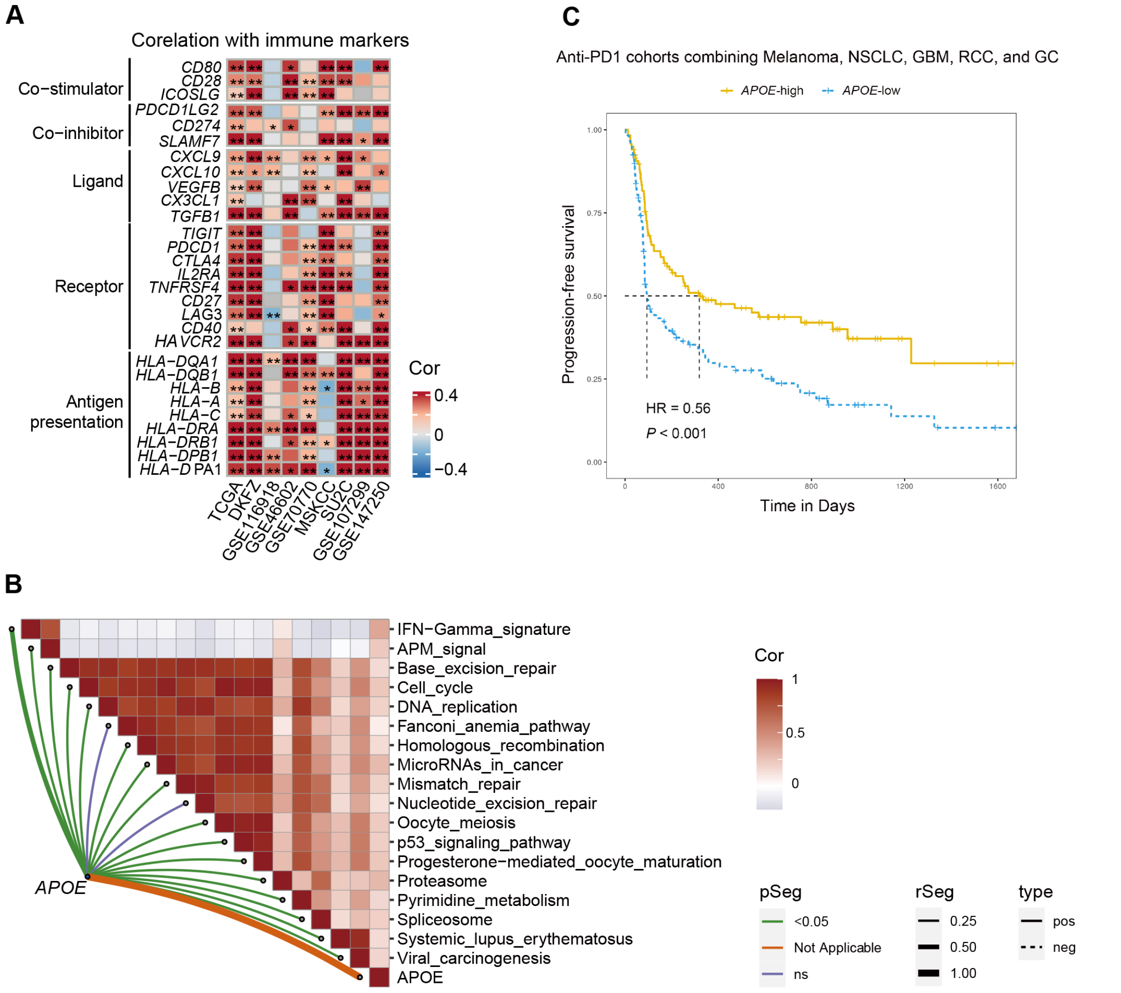

Figure S6: Correlation between APOE expression and the effectiveness of immunotherapy.

(A) A correlation analysis between APOE and immunoregulatory gene expression across multiple PCa cohorts.

(B) Established correlations between APOE expression and the enrichment scores of immunotherapy prediction pathways.

(C) Evidence from ICB atlas indicates a positive correlation between high APOE expression and improved PFS across anti-PD1 cohorts, incorporating melanoma, NSCLC, GBM, RCC, and GC cases.

*, $p < 0.05$; **, $p < 0.01$; ***, $p < 0.001$.

Abbreviations: ICI, immune checkpoint inhibitor; NSCLC, non-small cell lung cancer; GBM, glioblastoma, RCC, renal cell carcinoma; GC, gastric cancer

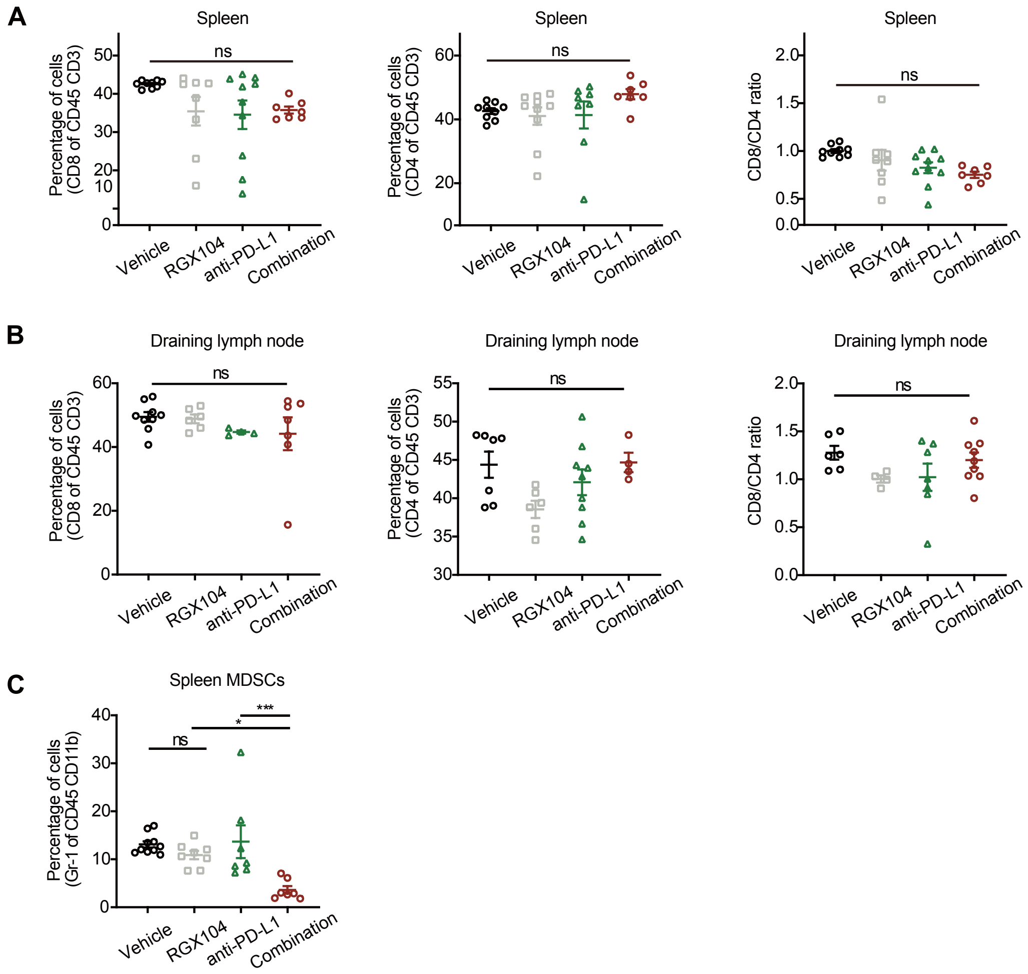

Figure S7: Association between APOE and immune characteristics in PCa

(A-B) Statistical analysis was performed to assess the differences in TILs across the indicated groups within the spleen (A) and draining lymph node (B).

(C) Quantitative analysis was conducted to examine MDSCs across specific groups within the spleen.

*, $p < 0.05$; **, $p < 0.01$; ***, $p < 0.001$.

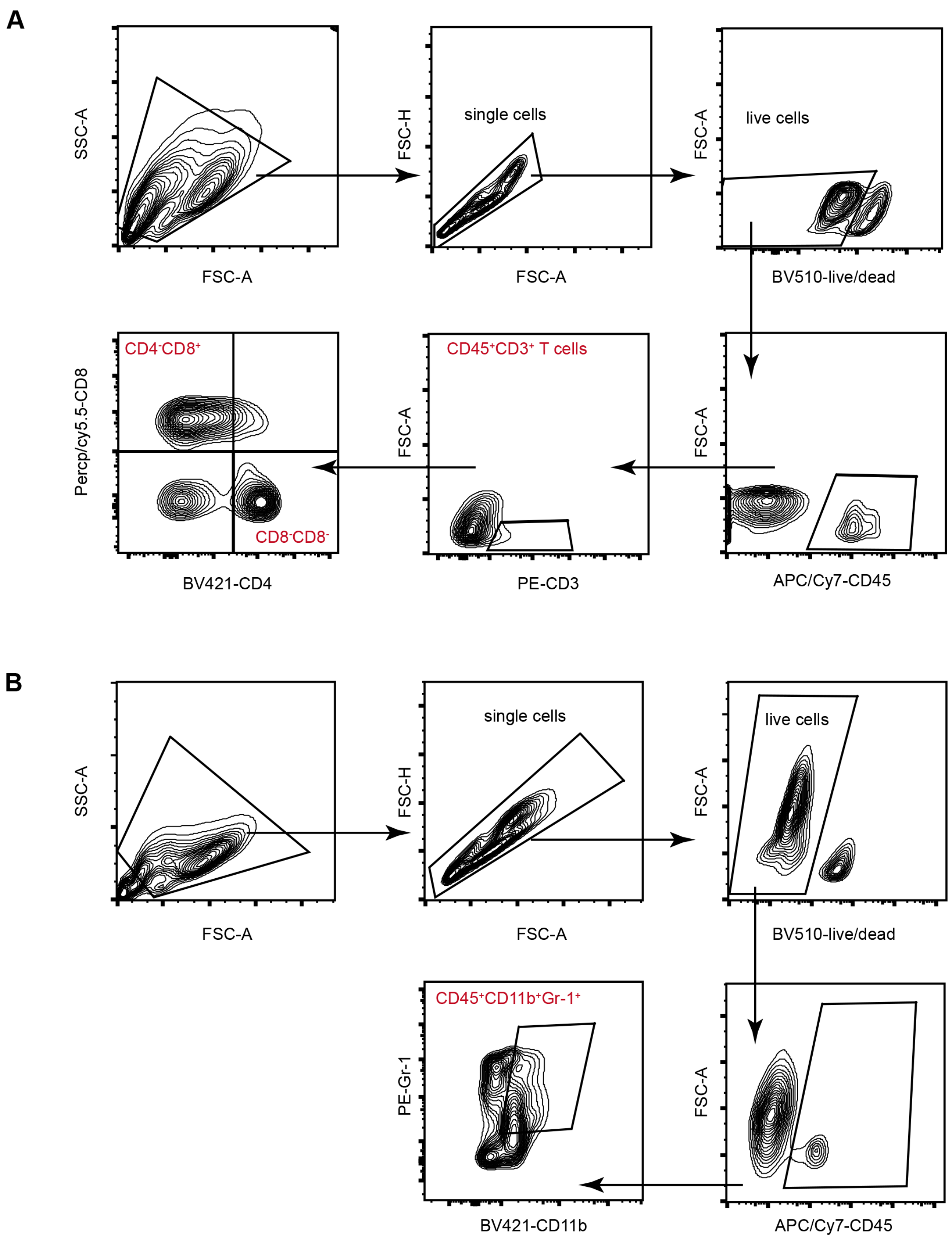

Figure S8: Gating strategies for TILs and MDSCs.

(A) Gating strategies for TILs.

(B) Gating strategies for MDSCs.

Abbreviations: TIL, tumor-infiltrating lymphocyte; MDSC, myeloid-derived suppressor cells.

Supplementary Methods

scRNA-seq data processing

The Novogene Tools pipeline was utilized to process the cleaned reads and assemble the transcript expression matrices. A rigorous quality control procedure was implemented for the cells, based on four principal metrics: the total count of unique molecular identifiers (UMIs), the number of genes detected, the percentage of mitochondrial gene counts relative to each cell, and the percentage of hemoglobin gene counts relative to each cell. The cell filtering criteria were as follows: exclusion of cells that (1) exhibited gene expression levels below the minimum threshold of 500 or above the maximum threshold of 4000, (2) demonstrated UMI counts below the lower limit of 1500 or surpassing the upper limit of 15,000, (3) contained more than 5% of their gene content derived from mitochondrial genes, or (4) possessed hemoglobin gene representation surpassing 0.1%. Beyond the routine processing steps, the "DoubletFinder" tool was employed to detect and remove potential doublets from each sample[1].

After rigorous quality control and the elimination of doublets, only high-quality single cells were selected for further analysis. These cells underwent analysis using the "Seurat" package[2], which was instrumental in creating gene expression matrices through the "NormalizeData" and "ScaleData" functions. The "Harmony" algorithm was then employed to integrate cells from various samples into a unified space[3]. After batch-effect correction, the top 30 principal components with the most significance were chosen for dimension reduction and visualization of gene expression using Uniform Manifold Approximation and Projection (UMAP) and t-distributed stochastic neighbor embedding (tSNE). The "FindClusters" function within the "Seurat" package was utilized to identify marker genes within each cluster. The initial clustering round, set at a resolution of 0.6, distinguished 6 cell types based on their distinct cellular markers.

Functional enrichment analysis and pathway activity score calculation

Differential expression analysis was conducted using the "FindMarkers" function from the Seurat package, adhering to the default settings. Genes were considered significantly differentially expressed (DEGs) if they met the criteria of p_val_adj < 0.05 and |avg_log2FC| > 0.2. Subsequently, the "clusterprofiler" R package was employed to perform Gene Ontology (GO) and Kyoto Encyclopedia of Genes and Genomes (KEGG) pathway analyses using the "enrichGo" and "enrichKEGG" functions, respectively, following the default parameters. The enrichment scores for a given gene signature of each cell were calculated using the R package "AUcell"[4]. A comprehensive list of genes utilized for calculating gene scores is provided in **Table S12**. Additionally, androgen receptor activity was estimated from gene expression data across samples using the "DoRothEA" R package[5].

Analysis of the human PCa dataset GSE141445

The dataset comprising 13 human PCa samples with single-cell RNA sequencing data (GSE141445) was employed as a validation cohort. The Count matrix files and corresponding metadata information were downloaded from the TISCH database[6]. We processed the data using the standard Seurat pipeline. Utilizing the standard Seurat pipeline, we processed this data. We specifically isolated malignant cells that exhibited discernible APOE expression and proceeded to calculate the enrichment scores for the AR response gene signature and the transcriptional activity of AR for each cell, as previously described.

1. McGinnis, C. S., Murrow, L. M. & Gartner, Z. J. DoubletFinder: Doublet Detection in Single-Cell RNA Sequencing Data Using Artificial Nearest Neighbors. *Cell Syst* **8**, 329-337.e4 (2019).
2. Hao, Y. *et al.* Integrated analysis of multimodal single-cell data. *Cell* **184**, 3573-3587.e29 (2021).
3. Korsunsky, I. *et al.* Fast, sensitive and accurate integration of single-cell data with Harmony. *Nat Methods* **16**, 1289–1296 (2019).
4. Aibar, S. *et al.* SCENIC: single-cell regulatory network inference and clustering. *Nat Methods* **14**, 1083–1086 (2017).
5. Garcia-Alonso, L., Holland, C. H., Ibrahim, M. M., Turei, D. & Saez-Rodriguez, J.

Benchmark and integration of resources for the estimation of human transcription factor activities. *Genome Res* **29**, 1363–1375 (2019).

6. Han, Y. *et al.* TISCH2: expanded datasets and new tools for single-cell transcriptome analyses of the tumor microenvironment. *Nucleic Acids Res* **51**, D1425–D1431 (2023).
